# Network medicine based approach for identifying the type 2 diabetes, osteoarthritis and triple negative breast cancer interactome: Finding the hub of hub genes

**DOI:** 10.1016/j.heliyon.2024.e36650

**Published:** 2024-08-22

**Authors:** Ilhaam Ayaz Durrani, Peter John, Attya Bhatti, Jahangir Sarwar Khan

**Affiliations:** a Atta-ur-Rahman School of Applied Biosciences, National University of Sciences and Technology, Islamabad, 44000, Pakistan; b Rawalpindi Medical University, Rawalpindi, Pakistan

**Keywords:** Type 2 diabetes mellitus, Osteoarthritis, Triple negative breast cancer, Meta hub genes, Bioinformatics, Gene mining, Network analysis, Switch genes

## Abstract

The increasing prevalence of multi-morbidities, particularly the incidence of breast cancer in diabetic/osteoarthritic patients emphasize on the need for exploring the underlying molecular mechanisms resulting in carcinogenesis. To address this, present study employed a systems biology approach to identify switch genes pivotal to the crosstalk between diseased states resulting in multi-morbid conditions. Hub genes previously reported for type 2 diabetes mellitus (T2DM), osteoarthritis (OA), and triple negative breast cancer (TNBC), were extracted from published literature and fed into an integrated bioinformatics analyses pipeline. Thirty-one hub genes common to all three diseases were identified. Functional enrichment analyses showed these were mainly enriched for immune and metabolism associated terms including advanced glycation end products (AGE) pathways, cancer pathways, particularly breast neoplasm, immune system signalling and adipose tissue. The T2DM-OA-TNBC interactome was subjected to protein-protein interaction network analyses to identify meta hub/clustered genes. These were prioritized and wired into a three disease signalling map presenting the enriched molecular crosstalk on T2DM-OA-TNBC axes to gain insight into the molecular mechanisms underlying disease-disease interactions. Deciphering the molecular bases for the intertwined metabolic and immune states may potentiate the discovery of biomarkers critical for identifying and targeting the immuno-metabolic origin of disease.

## Highlights

• Type 2 diabetes (T2D), osteoarthritis (OA) and triple negative breast cancer (TNBC) are immuno-metabolic diseases
• Present study employed bioinformatics analyses to identify eight hub/core genes central to T2DM-OA-TNBC interactome
• These meta hub genes may serve as potential targets for addressing multi-morbidity associations
• At the crux of this interactome, *AKT1-NFKB1* axis was found to be central to the meta-hub genes identified

## Graphical abstract

**Figure:**
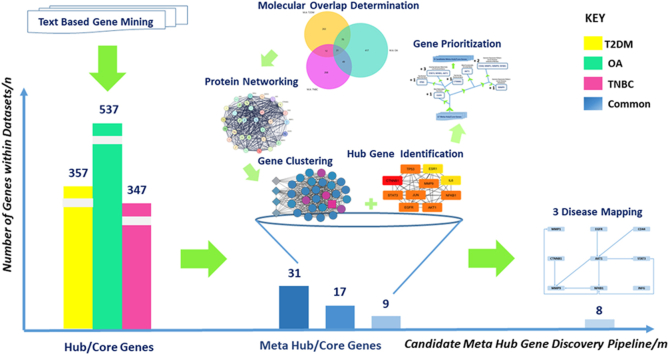

## 1 Introduction

The emerging field of network medicine is significantly contributing towards evolving human genomics, particularly in context to diseases. Molecular mysteries underlying polygenic complex patho-physiologies such as cancer and other non-communicable diseases are now being decoded with the analysis of high throughput omics data. Particularly, the study of gene-gene interactions and perturbations at cellular level, enable the construction of diseasomes and identification of their hub of interconnectivity, also termed as switch genes [1]. These hubs may serve central and critical roles in disease pathogenesis.

While the dynamics of these genes and their networks are context specific, in terms of expression, cellular or tissue localization and disease, yet they may exhibit a molecular overlap with associated conditions. For instance, there exists a risk modifying association between diabetes and several types of cancer, including breast tumorigenesis [2,3]. In yet another dimension, further complexities originate as both these diseases are associated with other complex diseases including but not limited to arthritis [[4], [5], [6], [7]]. Within the dimensions of this meta-diseasome underlying complex diseases and their interactions, lie the type 2 diabetes-osteoarthritis- breast cancer axes. In consequence, there is phenotypic emergence of multi-morbidity associated with inter-disease causative relationships such as type 2 diabetes mellitus (T2DM) and osteoarthritis (OA) serving as independent risk factors for BC [7,8] and T2DM associating positively with OA. This formulates the ground for finding a common origin of disease.

In line with this, several questions arise: to what extent do commonalities exist between these morbidities? How much of an overlap in disease signalling pathways and molecular mechanisms intersect and how does this potentiate an association? To address these, it is paramount to study disease characteristics individually and in combination, probe into disease progression mechanistic and model disease-disease interactions.

This phenomenon of inter-disease relationships is supported by surmounting epidemiological evidence. T2DM is a common metabolic disorder, developing as a growing pandemic with a global prevalence of 476 million in 2017 and projected to rise to 570.9 million by 2025 [9]. To add to this burden on public health concern, it is reported that T2DM patients are at a moderate yet significantly elevated risk of developing BC [10]. Furthermore, patients, in particular those taking metformin in long-term, are at a 38 % elevated risk of developing a specific subtype; triple negative BC (TNBC) [11]. TNBC accounts for 10–15 % of all reported BC cases [12]. It is characterized by a lack of expression of *estrogen* *receptor* *(ER),* *progesterone* *receptor* *(PR)* *and* *human* *epidermal* *growth* *factor* *2* *(HER2)*, translating into an aggressive phenotype and poor survival outcome. TNBC is a heterogeneous disease, itself, stratified into various subtypes, emerging from an interplay of genetic and environmental variables [13].

While, OA is predominantly an inflammatory disease affecting multiple joints, with the foot OA at a prevalence of 16.7 % for adults aged fifty years and older and knee OA at 16 % worldwide [8,9]. Furthermore, the association between OA and BC, although not as well defined as the T2DM-(TN)BC relationship, and still critically under-explored, is also reported [7]. Moreover, T2DM patients are more susceptible to developing OA [4], The focus of this research is to study these three diseases together to gain insight into a potentially common origin of disease and also, particularly, to unravel the molecular interplay underlying T2DM and OA as risk factors for TNBC.

Breast carcinogenesis is a complex, multifactorial and heterogeneous phenomenon implicating genetic drivers and environmental triggers resulting in an intricately regulated cascade of aberrant cell signalling in mammary tissue. This orchestrates molecular mechanisms underlying hallmarks of cancer including but not limited to abnormal cellular growth, evasion from apoptosis, over-activation of immune pathways leading to tumor promoting inflammation and dysregulated cellular genetics.

At genetic level, this may be onset by susceptibility/risk factors including mutations in genes such as breast cancer gene 1 (*BRCA1)* which are associated with an increased risk of developing (TN)BC [14]. At meta level, disease-disease interactions emerging from molecular crosstalk implicating switch genes may also result in multi-morbid state.

The superimposition of risk factors including but not limited to genetic predisposition and lifestyle choices such as diet and physical activity, and their synergy with conditions including aging, obesity and dyslipidemia potentially formulate the molecular bases underlying the multifaceted association between these three morbidities. Evaluating these mechanics may provide elucidation on the converging phenotypic emergence of signature characteristics such as chronic inflammation, oxidative stress, metabolic alterations and hormonal imbalances, and their underlying molecular dialogue, presenting varying degrees of complexities and interconnected diseased states [15,16]. Recent studies explore the role of immune cells in modulating metabolic homeostasis [17]. In particular, immune cell activation is reported to trigger metabolic reprogramming of anaerobic glycolysis, oxidative phosphorylation, and metabolite synthesis [18,19]. Conversely metabolism sponsors the energy demands for all cellular functions including immune response and inflammatory pathways, creating an immuno-metabolic feedback loop. However, this is not the extent of crosstalk between these two states, which unravels into a multi-faceted, multi-layered, and intricately related integration of molecular communication [20,21]. At the next level, alterations in metabolic pathways further drive immune-modulatory mechanisms underlying innate and adaptive immunity [21]. Hence, this critical equation is bidirectional and may determine cellular fate. At the union of these two states, chronic metabolic inflammation, referred to as ‘metaflammation’ is reportedly a basis for disease development and a known hallmark for metabolic disorders.

Hence, the interplay between the onco-characteristics on the spectrum of polygenic phenotypes, particularly implicating the crosstalk between metabolic rewiring mechanisms and chronic inflammatory feedback loops short circuit into a cluster of diseases including complex patho-physiologies beyond cancer, all mapped by common pathways and overlapping regulatory networks.

To investigate this phenomenon in context to the origin of complex disease, present work employed network biology approach to identify common genetic players between the three complex morbidities. These genes may serve as potential biomarkers for disease initiation, early secondary disease diagnosis in case of bi-morbidity, prognostic prediction and therapeutic targeting. To the best of our knowledge, this is the first instance where T2DM, OA and TNBC have been grouped together as common immuno-metabolic diseases to investigate mutually inclusive genes and pathways. In particular, the objective is to understand the underlying the molecular mechanisms associated with these complex etiologies, in pursuit of decoding the immuno-metabolic origin of disease. Since common and complex diseases such as these three may involve hundreds of genes with smaller effects on disease pathogenesis, rather than singular or fewer genes with more deleterious effects in case of rare disorders, network biology has enabled the study of diseasomes to identify hub genes prevalently dominating signalling pathways and molecular crosstalk within a functional unit. Identification of these hub genes, through in silico analysis of high throughput data generated large gene-gene/protein-protein interaction networks may provide potential biomarkers associating with disease pathogenesis. On the next level, super-imposing disease specific hub genes may allow for the emergence of common development patterns for the origin of disease, ultimately highlighting the highway to immuno-metabolic disease initiation and the route for its blockade potentiating disease prevention and treatment, and specifically, precision medicine.

## 2 Materials and methods

### 2.1 Article selection

**Fig. 1:**
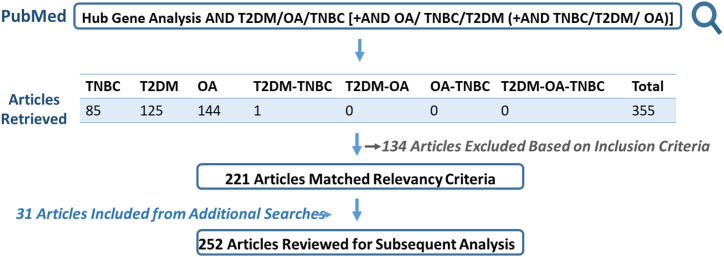
**Literature search and article selection.** PubMed database was searched for articles on hub gene analysis for type 2 diabetes mellitus (T2DM), osteoarthritis (OA), and triple negative breast cancer (TNBC) in singularity and in combination. A total of 252 were henceforth utilized for subsequent disease specific hub/key genes mining.

A comprehensive literature review search was done by utilizing the keywords “hub gene analysis” AND “type 2 diabetes mellitus” AND/OR “triple negative breast cancer” AND/OR “osteoarthritis” on PubMed repository to retrieve a total of 355 articles, combined from individual string searches. PubMed was utilized as it provides a reliable publicly available interface for searching medicine and health related literature [22]. Screening on the basis of relevance concluded with 221 articles along with 31 additional articles meeting the selection criteria as defined subsequently. Articles from the time period of 2012–2023, designed on identifying hub/key/core genes through bioinformatics, implicated in any of these three diseases or in cross disease comparison, were included in this study. Hence, 252 articles were resourced for text based gene mining for subsequent analysis. These articles included the derivation and network analysis of differentially expressed genes from publicly available microarray based transcriptomic datasets. Fig. 1 outlines the literature search process.

### 2.2 Bio-computational framework implementation

**Fig. 2:**
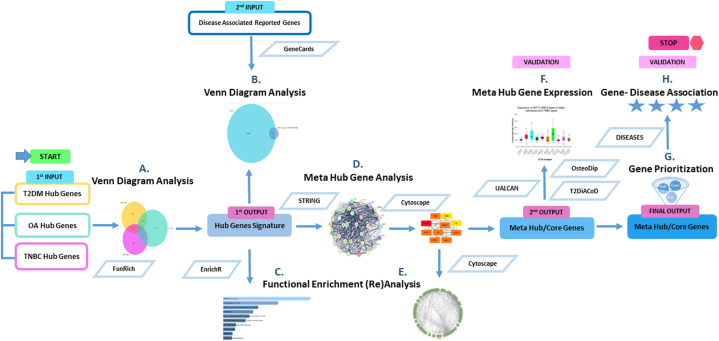
**Study methodology flow diagram.** The disease specific hub/core genes were input into the candidate meta hub gene discovery pipeline to generate potential multi-morbidity associated biomarkers. Start and end point are indicated and input/output are referred to in terms of genes. The outlined rectangle boxes represent input, blue outlined rhombus-software/tool/database, light blue coloured box-critical outcome/secondary input, medium blue box-intermediary output and dark blue box-final output/outcome in terms of critical genes. (For interpretation of the references to colour in this figure legend, the reader is referred to the Web version of this article.)

This research work utilized text based mining of hub/core genes for three complex diseases of immuno-metabolic origin, intended as input for a series of integrated bioinformatics analyses. The subsequent outcome may potentiate multi-morbidity associated biomarker discovery for the molecular origin of disease in metabolic disorders such as T2DM, OA and TNBC. Fig. 2 outlines the methodology framework adopted for this study.

#### 2.2.1 Text based gene data retrieval

Shortlisted research articles were manually screened thoroughly to enlist hub genes identified for each of the three diseases. These lists were stored as individual training datasets for subsequent analyses (Fig. 2).

#### 2.2.2 Venn diagram analysis

Disease specific hub genes' lists were imported into FunRich software to determine the molecular overlap between the T2DM, OA and TNBC, as shown in Fig. 2A. FunRich is an open access multipurpose bioinformatics tool, commonly used for analysing large gene datasets [23]. It provides a user friendly solution for Venn diagram analysis. The common genes’ list was retrieved as a multi-morbidity associated signature molecular profile.

#### 2.2.3 Phenotype to gene mapping

Gene data corresponding to all three diseases, their hallmarks and other characteristic features were downloaded from GeneCards database (https://www.genecards.org/), and an overlap of each of the datasets with the common hub genes was determined using FunRich (Fig. 2B). GeneCards is a comprehensive repository of human expressed genes associating with diseases, phenotypes and biological pathways [24]. It provides a combined interface for gene-disease-pathway links, hence it was opted for disease/phenotype associated gene mining.

#### 2.2.4 GO and pathway enrichment

The molecular overlap i.e the common hub genes’ list was imported into EnrichR (https://maayanlab.cloud/Enrichr/) to perform gene ontology (GO) and pathway enrichment (PE) analyses (Fig. 2C). EnrichR is an online interactive gene set enrichment analysis (GSEA) tool, routinely used for GO and Kyoto Encyclopedia of Genes and Genome (KEGG) analyses [25]. Compared to other enrichment analyses tools, EnrichR provides a user friendly interface, and a comprehensive analysis with interactive visualization. Top enrichment terms extracted from various embedded libraries are visualized based on adjusted *p* value < 0.05.

#### 2.2.5 PPI network construction, gene clustering and meta hub genes identification

The common hub genes list was uploaded onto STRING version 12.0 (https://string-db.org/) to generate a protein-protein interaction (PPI) network and then imported into Cytoscape version 3.10.1 to analyse its network topological parameters (Fig. 2D). Molecular complex detection (MCODE) plugin (v2.0.3) was applied to identify core module genes, using default settings. MCODE identifies densely connected components of the PPI network representing molecular complexes [26]. CytoHubba plugin (v0.1) were employed to determine the meta hub genes. This plugin applies 11 network topological analyses to determine the important nodes of a network.

#### 2.2.6 Functional enrichment analysis of meta hub and core genes

Using Cytoscape plugin ClueGO (v2.5.10), functional enrichment analysis of the meta hub genes was performed for the three GO categories and KEGG pathways (Fig. 2E). ClueGO integrates GO terms to present functionally classified networks, which can be interpreted for the genes shared, determining the association between the over-represented terms based on kappa statistics [27]. It also provides plausible insights into pathway based networks for the identification of potential biomarkers. It performs the Fisher exact test for *p* value calculation and Bonferroni step-down method for multiple testing correction as the default option [28].

#### 2.2.7 Expression validation of meta hub genes

The expression of the hub genes with reference to each of the three diseases were retrieved from disease specific databases. These included T2DiACoD (https://t2diacod.igib.res.in/index.php), an online gene atlas for T2DM associated complex disorders with information from GEO datasets embedded within the website [29]. It applies Empirical Bayes moderated t-statistics test and Benjamini-Hochberg for adjustment of *p* value [30]. Additionally, a previously analysed T2DM adipose tissue expression dataset GSE29231 was also utilized (3 biological samples with 4 technical replicates each for T2DM as well as for healthy controls), using the cut off criteria of adjusted *p* value < 0.05 (Benjamini and Hochberg adjustment test) and |Log(FC)| ≥ 1 [31]. OsteoDip (http://ophid.utoronto.ca/OsteoDIP), a web based database for genes associating with OA which derives information from GEO series [32], and UALCAN (https://ualcan.path.uab.edu/index.html), a publicly available, online tool storing a comprehensive repository for gene expression data for up to 31 cancers and their subtypes, including (TN)BC (Welch T test for statistical difference) [33,34], were also utilized (Fig. 2F).

#### 2.2.8 Gene prioritization to determine master meta hub genes

The meta hub genes were subjected to gene prioritizing methods based on seven parameters for shortlisting candidates, and the outcome of core meta genes were listed (Fig. 2G).

#### 2.2.9 Gene-disease association

Association of each of the prioritized meta-hub genes with diseased state were confirmed on Diseases database (https://diseases.jensenlab.org/), Fig. 2H. Diseases is a regularly updated web source with integrated information on gene-disease associations based on multiple sources searched through automatic and manual text mining [35].

## 3 Results

### 3.1 Text based gene mining

**Fig. 3:**
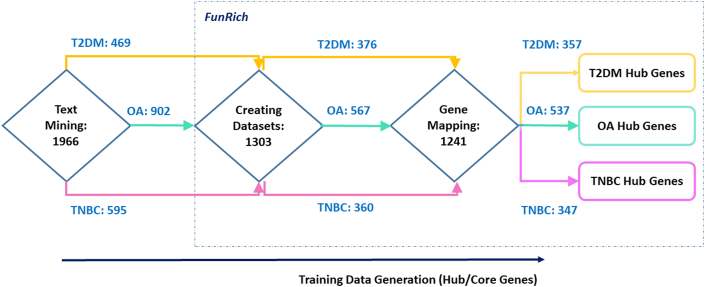
*Textminingandtrainingdatageneration. Hub/key/coregenesassociatingwith* type 2 diabetes mellitus (*T2DM)/osteoarthritis (OA)/triple negative breast cancer (TNBC)wereextractedfrompublishedliteratureandmappedontoFunRichsoftware.Statisticalfiguresindicatethenumberofgenesobtainedateachstep.*

Each article was carefully screened and hub genes (alternatively key/core genes) were extracted for all three diseases, as outlined in Fig. 3.

Each of these lists were mapped onto FunRich software to obtain the identified number of genes. Redundancy within the same gene list was removed by defining gene datasets. A total of 376, 567 and 360 genes were mined for T2DM, OA and TNBC respectively. These genes were then further subjected to a series of integrated bioinformatics analyses to explore their functional niche and potential implications within the T2DM-OA-TNBC axes.

### 3.2 Implementation of integrated bioinformatics analyses pipeline

#### 3.2.1 Identification of the molecular overlap between T2DM, OA and TNBC hub genes

**Fig. 4:**
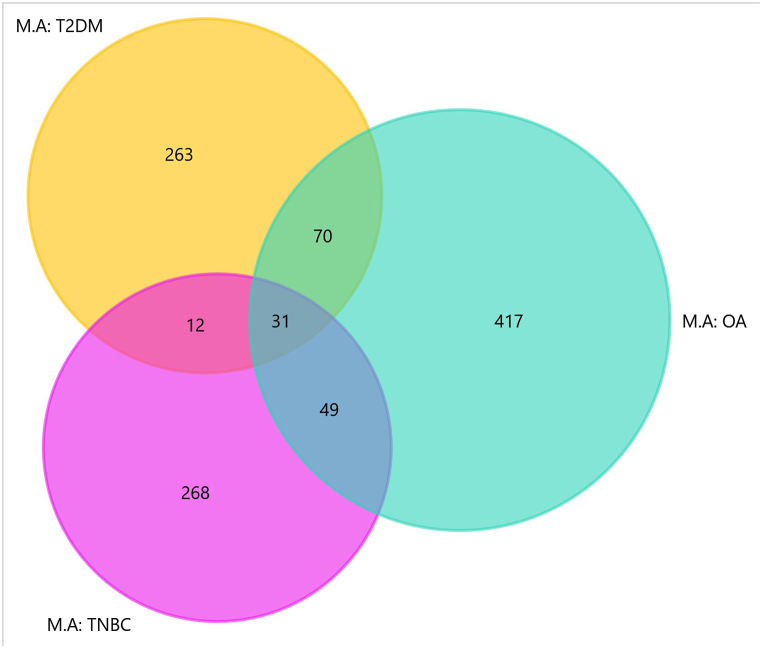
**Venn diagram analysis. T**he yellow circle represents type 2 diabetes mellitus (T2DM) hub genes derived for meta hub gene analysis (M.A), sea green-osteoarthritis (OA) and pink-triple negative breast cancer (TNBC). The intersection between circles represent overlapping genes common to more than one disease. A total of 31 genes overlapped between all three datasets. (For interpretation of the references to colour in this figure legend, the reader is referred to the Web version of this article.)

The meta-computational analysis involved the deciphering of the hub/key genes commonly implicated in all three diseases. For this, a Venn diagram representing an overlap between the training gene lists was generated, as shown in Fig. 4.

The intersections represented molecular overlap between all three datasets with the common hub genes identified in Table 1.

**Table 1:** **Molecular overlap betweentype 2 diabetes mellitus (T2DM),osteoarthritis(OA)andtriple negative breast cancer (TNBC).** A total of 31 genes are identified as the signature common hub genes between the three diseases.

Morbidity	No. of Common Genes	Genes
T2DM- OA- TNBC	31	*LCK;STAT1;IFNG;BIRC5;FN1;CD44;CTNNB1;TP53;CCND1;EGFR;ESR1;STAT3;KDR;JUN;MYC;AR;IL6;MMP1;MMP9;FOS;KRAS;UBC;IGF1R;AKT1;NFKB1;ITGB1;MAPK14;SPP1;MMP2;VCAN;RELA*

#### 3.2.2 Determination of molecular overlap representing phenotype to gene mapping

**Fig. 5:**
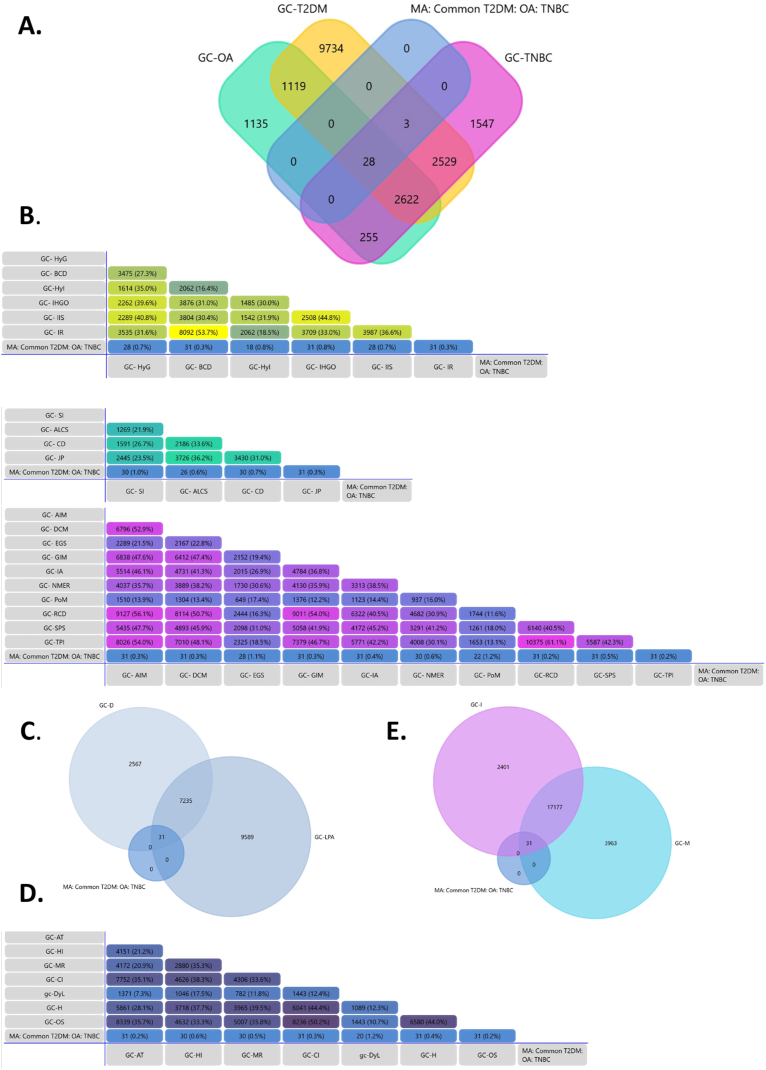
**Phenotype to gene mapping.** Overlap of 31 hub genes signature with GeneCards derived genes for A- T2DM, OA and TNBC, B- Hallmarks for T2DM (top), OA (middle), and TNBC (bottom), **C**- Common factors, **D**- Common characteristics and **E-** Common pathways. Abbreviations: M.A-meta hub gene analysis; T2DM-type 2 diabetes mellitus; OA-osteoarthritis; TNBC- triple negative breast cancer; GC- Gene Cards; HyG-hyperglycemia; BCD-beta cell dysfunction; HyI- hyperinsulinemia; IHGO- increased hepatic glucose output; IIS- inadequate insulin secretion; IR-insulin resistance; SI- synovial inflammation; ALCS- asymmetric loss of cartilage space; CD-chondrocyte degradation; JP- joint pain; AIM-activating invasion and metastasis; DCM-deregulating cellular metabolism; EGS- evading growth suppressors; GIM-genome instability and mutation; IA-inducing angiogenesis; NMER-non mutational epigenetics reprogramming; PoM-polymorphic microbes; RCD-resisting cell death; SPS- sustaining proliferative signals; TPI- tumor promoting inflammation; AT-adipose tissue; HI-hormonal imbalance; MR-; CI- chronic inflammation; H-; OS- oxidative stress.

Genes associating with each of these diseases, their phenotypic hallmarks, factors, molecular pathways and various other features were retrieved from GeneCards. For each of the corresponding gene lists, their overlap with the 31 hub genes' signature was determined in FunRich, as depicted in Fig. 5.

A significant overlap between various aspects of disease pathophysiology and its molecular mechanisms, particularly on the immuno-metabolic axis, amongst others was evident. This axis is well defined as the metabolic dysregulations, which are hallmark to T2DM, primarily a metabolic disorder, are also prevalent in both OA and TNBC, and each of these three diseases are attributed by chronic inflammation.

The union of genes specific to all three diseases from GC overlapped with 28 genes from the common hub genes signature (Fig. 5A). Fig. 5B depicts the molecular overlap between 31 hub gene signature and genes associating with disease specific hallmarks, followed by other features and terms studied, Fig. 5C–E. All 31 genes were shown to associate with beta cell dysfunction, increased hepatic output, insulin resistance, joint pain, tumour promoting inflammation, sustaining proliferative signals resisting cell death, inducing angiogenesis, genome instability and mutation, deregulating cellular metabolism and activating invasion and metastasis. Complete overlap was also found with diet, lack of physical activity, adipose tissue, chronic inflammation, hypoxia and oxidative stress associated genes.

#### 3.2.3 Gene functional enrichment

**Fig. 6:**
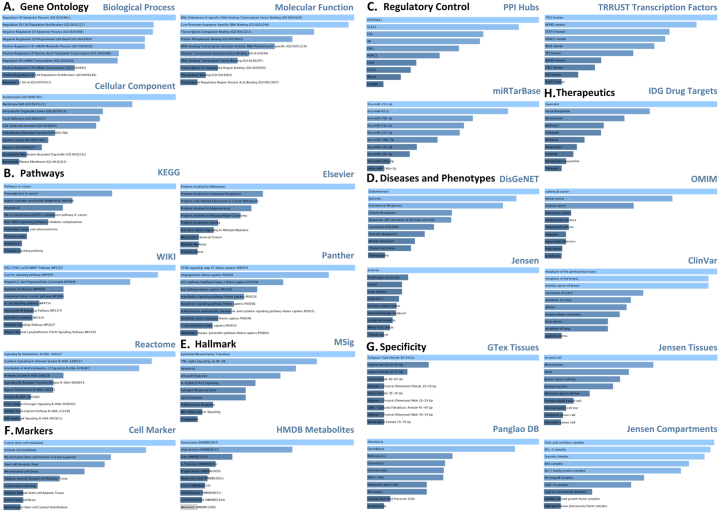
**EnrichR based functional enrichment analysis.***Enrichmentforvariouscategoriesaredepicted.****A****-Geneontologyincludingthreecategories,****B****-Pathways(fivesources),****C****-Regulation(threecategories),****D****-Diseasesandphenotypes(fourdatabases),****E****-Hallmark(onesource),****F****-Markers(twotypes),****G****.Specificity(fourcategories)and****H****-Therapeutics(basedon* one *database).*

The common hub genes were imported to EnrichR to perform functional enrichment analyses including GO and pathway enrichment. Most statistically significant results are displayed in Fig. 6.

##### 3.2.3.1 Gene ontology

For gene ontological assessment, statistically significant terms (adjusted *p* value < 0.05) including biological processes (BP), molecular function (MF) and cellular component (CC) were determined. Analysis outcome (Fig. 6A), displayed that the common hub genes are most significantly enriched with BP terms such as regulation of apoptotic (particularly negative) and other programmed cell death mechanisms, cell population proliferative processes, and positive regulation of miRNA implicated in metabolic processes. These genes were enriched for molecular functions including binding activities such as transcription factor binding of RNA polymerase II on DNA, transcription co-regulation and protein phosphatase binding. Furthermore, the most significantly enriched cellular component terms included euchromatin, membrane raft and intracellular organelle lumen.

##### 3.2.3.2 Pathways

Pathway enrichment analysis was conducted in accordance with integrated KEGG, Wiki, Reactome, Elsevier and Panther databases. The top significantly enriched terms are presented in Fig. 6B. The findings from each of these databases were comparable with a general theme of enrichment in pathways involved in cancer, particularly breast cancer, immune pathways and metabolic pathways. More specifically prolactin signalling and AGE-RAGE signalling (in diabetic complications), were presented by KEGG and WikiPathways sources, and *estrogen* and *ESR* signalling according to Reactome, amongst the most significant terms. Additionally, the Wiki pathway involving *RAC1/PAK1*'s implication in cell proliferation, angiogenesis and tumour growth, and the Panther pathways for inflammation, oxidative stress and apoptosis signalling were also listed.

##### 3.2.3.3 Regulatory control

Moreover, these genes were shown (Fig. 6C) to associate with enriched PPI hub genes such as *HSP90AA1,* *STAT3,* *FOS,* *ESR1,* *EGFR* and *BRCA1*, majority of which are already existent within the hub genes derived interactome under study. The transcription factor analysis based on information from integrated TRRUST database, revealed significant enrichment for *TP52,* *NFKB1,* *STAT3,* *ESR1* and *JUN* amongst others. The top three significantly enriched miRNAs derived from miRTarBase included hsa-miR-155-5p, hsa-miR-451a, and hsa-miR29b-3p.

##### 3.2.3.4 Diseases and phenotypes

In addition, the association of the sub interactome with disease and phenotypic terms were also studied based on several databases integrated within the EnrichR interface (Fig. 6D). According to DisGeNET, the 31 hub genes based interactome was implicated in endometriosis and various cancer types including osteosarcoma. This was reiterated by the resulting output from OMIM Diseases which included breast cancer and additionally rheumatoid arthritis amongst the most enriched terms. Jensen Diseases also showed enrichment for cancer types including cancer of the immune system amongst others and more general terms like cancer and arthritis. Similarly, ClinVar also displayed breast neoplasms, particularly familial cases amidst other cancers as the most significantly enriched terms.

##### 3.2.3.5 Hallmark

For the enrichment within hallmark terms, results derived from MSig database, as displayed in Fig. 6E, showed significance for terms including epithelial mesenchymal transition (EMT), a characteristic hallmark of breast cancer, but also interestingly, of OA pathogenesis and diabetic complications such as diabetic kidney disease*,* *TNF-alpha* signalling via *NFKB*, apoptosis and inflammatory response.

##### 3.2.3.6 Biomarkers

The interactome under study particularly associated with terms such cancer stem cell, mesenchymal cells and adipose derived stromal and stem cell, with statistical significance (Fig. 6F). Their association with metabolites was also determined through the EnrichR analysis retrieving information from the HMDB metabolites database. Amongst the most significantly enriched terms, Simvastatin, aldosterone and Zinc were listed.

##### 3.2.3.7 Specificity

These hub genes were also found to be present mainly in visceral adipose tissue and whole blood, according to GTex tissues, stromal cell, breast cancer cell line, mammary gland cell line, immune system (Jensen tissues), fatty acid synthase complex, BCL-2 complex and NFKB complex (Jensen compartments). Furthermore, fibroblasts, osteoblasts, osteoclasts, chondrocytes, Miller cells, monocytes and stromal cells (PanglaoDB), and smooth muscle, adipocyte, pancreatic islet and various types of immune cells based on Human gene atlas, were also included as some of the most significantly enriched terms (Fig. 6G).

##### 3.2.3.8 Therapeutics

Enriched drug targets were determined and the top three most significant terms included Quercetin, Hexachlorophene and Niclosamide, according to the embedded IDG drug targets source, Fig. 6H.

#### 3.2.4 Hub gene network analysis

##### 3.2.4.1 Construction of protein-protein interaction network

Subsequently, the 31 common hub genes were entered onto the STRING search interface to generate a protein-protein interaction (PPI) network with 31 nodes, 379 edges and an average node degree of 24.5 (PPI interactions score ≥ 0.4), as shown in Supplementary Fig. 1.

##### 3.2.4.2 Identification of clustered genes

**Fig. 7:**
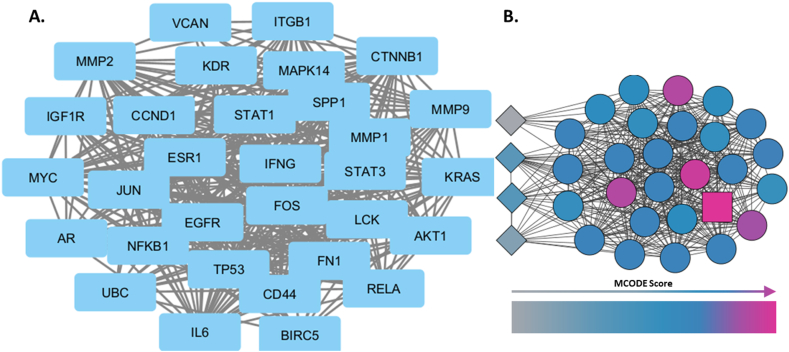
**Cytoscape based network analysis. A***-****A****.OriginalnetworkimportedfromSTRINGwithnodesrepresentedbylabelledbluerectanglesandedgesbygreylines.****B***- *Cluster1derivedthroughapplicationofMCODEplugin,withnodesrepresentedbyrhombusasun-clustered,circleasclusteredandrectangleasseed.TheincreaseinnodecolourintensityrepresentsincreasingMCODEscore.* (For interpretation of the references to colour in this figure legend, the reader is referred to the Web version of this article.)

The topological network as visualized on Cytoscape is depicted in Fig. 7A. MCODE plugin was applied and network analysed to determine clusters within the network. As demonstrated in Fig. 7(B), 1 cluster was found comprising of 27 nodes, with a score of 25.077. Top 10 genes from within the cluster, with the highest MCODE scores were determined and listed in Supplementary Table 1. *IFNG* was found to be the seed of the cluster.

##### 3.2.4.3 Identification of meta hub genes

Hub gene analysis was performed based on multiple algorithms implemented by the Cytohubba plugin of Cytoscape, as detailed in Supplementary Table 2. The top 10 genes according to each of the methods were extracted alongside their ranks. *CTNNB1* was ranked to be the highest according to a significant majority of the methods including Maximum Clique Centrality (MCC), Degree, Betweeness, Closeness and Maximum Neighborhood Component (MNC), followed by *STAT3,* *TP53,* *EGFR,* *NFKB1,* *JUN,* *MMP9,* *AKT1,* *IL6* and *ESR1*. These final top 10 meta hub genes were identified based on the frequency of their occurrence within the top rankings.

#### 3.2.5 Pathway enrichment reanalysis of meta hub genes

**Fig. 8:**
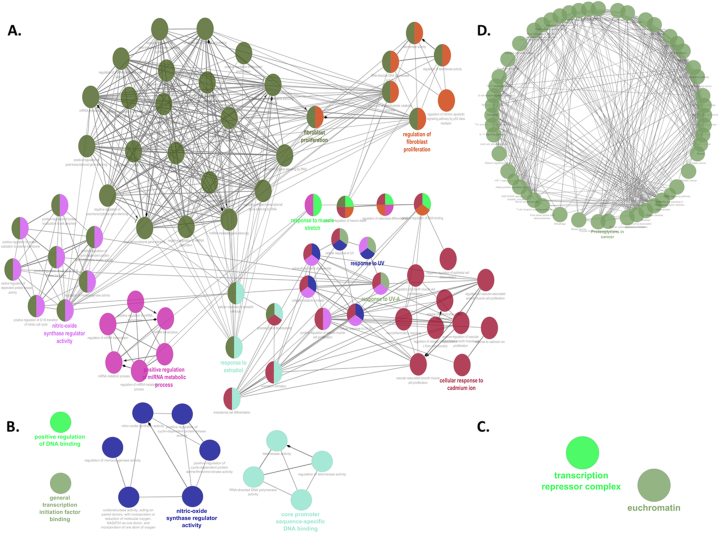
**Gene ontology and pathway reanalyses on Cytoscape.** The nodes represent different terms, their colours correspond to functional groups and the edges between nodes indicate interaction and crosstalk. Nodes with multiple colours symbolize genes common between more than one term. **A**- Gene ontology (GO) biological processes, **B**- GO molecular function, **C**- GO cellular component and **D**- KEGG pathway. (For interpretation of the references to colour in this figure legend, the reader is referred to the Web version of this article.)

The top 10 hub genes were combined with the top 10 clustered genes, to generate a list of 17 meta hub/core/genes, with a molecular overlap of 3 genes. The functionality of these genes was studied by re-performing the GO and pathway analyses using Cytoscape plugin ClueGO. The results are displayed in the form of functionally clustered networks, Fig. 8.

The GO reanalysis of the derivative sub-interactome (Fig. 8A–C), showed the genes to be enriched with BP terms including positive regulation of miRNA metabolic process (pink), fibroblast proliferation (dark green) and its regulation (orange), response to UV (blue) and cadmium (maroon), nitric oxide synthase regulator activity (purple) and muscle stretch (light green). Furthermore, osteoclast differentiation, response to estradiol, extracellular matric disassembly, release of mitochondrial cytochrome C, regulation of carbohydrate catabolic process and regulation of intrinsic apoptotic signalling pathway for p53 class mediator are amongst other significant and clustered BP terms, highlighting the biological programming of the meta hub genes’ network. The significantly enriched MF terms for these genes included positive regulation of DNA binding (bright green), transcription initiation factor binding (green), nitric oxide synthase regulator activity (royal blue) and core promoter sequence specific DNA binding (cyan). Moreover, there were two CC terms; transcription repressor complex and euchromatin, reflecting at the critical involvement of the meta hub genes in gene expression and its regulation.

The KEGG pathway analysis, as depicted in Fig. 8D, revealed enriched terms such as chemical carcinogenesis, choline metabolism in cancer, estrogen signalling, AGE/RAGE signalling, lipid and atherosclerosis, central carbon metabolism in cancer, *HIF1* signalling, adipocytokine signalling pathway, mitophagy, and various terms related to cancer types, particularly including breast cancer, and immune signalling pathways including *TNF* signalling and B cell and T cell pathways, amongst others, with proteoglycans in cancer being the most significant.

#### 3.2.6 Validation of meta hub genes expression

**Fig. 9:**
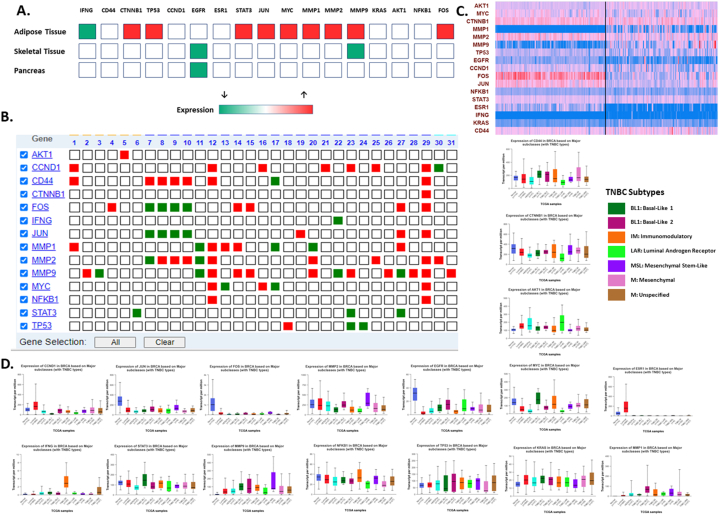
**Disease specific meta hub gene expression. A-** T2DiACoD database and GEO dataset GSE29231 generated expression data for type 2 diabetes mellitus (T2DM). The heat map depicts tissue specific expression for T2DM, p < 0.05, red: up-regulated; green: down-regulated, **B-** OsteoDip generated data expression for osteoarthritis (OA). The snapshot represents heat map generated for OA specific gene expression for meta hub genes based on 31 sources, red: up-regulated; green: down-regulated, **C-** UALCAN generated expression data for triple negative breast cancer (TNBC). The generated heat map for the meta hub genes show the expression pattern of each of the genes across TNBC and healthy control samples, **D**- TNBC subtype specific expression data for meta hub genes. BRCA: Breast invasive carcinoma. (For interpretation of the references to colour in this figure legend, the reader is referred to the Web version of this article.)

To study the disease specific expression of each of the meta hub genes, various specialized databases were utilized. T2DM specific gene expression data corresponding to meta hub genes was derived from T2DiACoD and GEO Series GSE29231 as shown in Fig. 9A.

Meta hub/core genes such as *CTNNB1,* *TP53,* *STAT3,* *JUN,* *MYC,* *MMPs* and *FOS* were found to be up-regulated in T2DM adipose tissue. Expression data specific to OA for these meta hub genes was retrieved from OsteoDip. The 17 meta hub genes were fed into the server which returned a heat map profile for 14 genes as shown in Fig. 9B (expression data for *KRAS,* *EGFR* and *ESR* was not found). The database showed expression profiles from 31 sources, 6 of which were disease vs. disease, 2 were evaluative and the remaining were disease vs. normal. The up-regulated genes included *CCND1,* *CD44*, and *NFKB1*, however not across all samples. Furthermore, expression data for TNBC was derived from UALCAN database. A heat-map for the 17 meta hub genes were generated, as shown in Fig. 9C. TNBC subtype specific data for each of these genes was also studied, particularly for the immunomodulatory subtype and is presented in Fig. 9D. Up-regulated genes included *IFNG,* *STAT3* and *MMP9*.

In further support of this, expression status of each gene for all three diseases was retrieved from experimental data and published literature. Supplementary Table 3 presents a summary of their expression pattern across the three diseases. Additionally, as another measure towards validation, the redundancy of occurrence within the parent training datasets for each of the meta hub genes was evaluated and is indicated in Supplementary Table 4.

#### 3.2.7 Gene prioritization

**Fig. 10:**
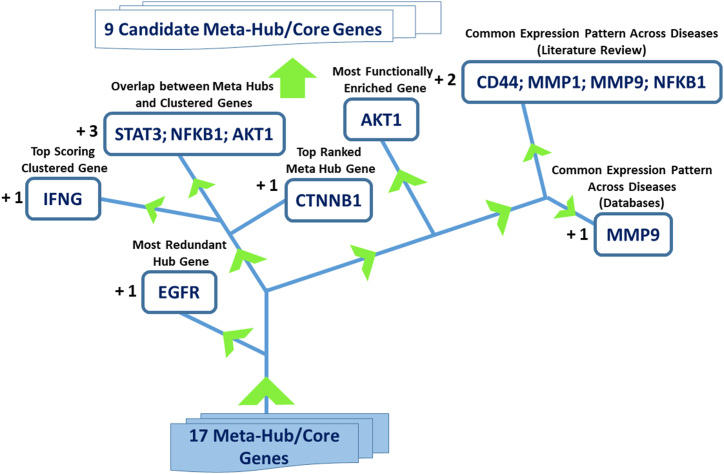
**Gene prioritization.** Various parameters were applied to shortlist 9 meta hub genes.

To capture the bio-contextual highlight of this interactome, hub of hub genes prioritization was exercised based on several parameters: (1) redundancy within the parent hub gene list derived from text mining, (2) ranking based on hub gene- and (3) MCODE analysis, (4) inclusion within the molecular overlap between meta hub genes and clustered/module genes list, (5) frequency/degree of enrichment of genes in functional enrichment analysis, and similar expression pattern- (6) across the three diseases based on databases, and (7) literature search. For each of these parameters, the best performing gene was shortlisted, except in the case of molecular overlap between meta-hub and clustered genes which comprised of 3 genes and common expression pattern exhibiting genes which presented with an addition of 2 genes. The final derivation of the shortlisted candidate meta hub genes, 9 in total, is diagrammatically represented in Fig. 10.

#### 3.2.8 Confirmation of gene-disease association

The gene-disease association for each of the candidate meta hub genes was assessed on Diseases database, which assigns a z score against a confidence level for genes associating with a disease term based on text mining, Supplementary Table 5. Each gene was assigned a confidence based on the source of the association, with the automatic text mining capped at 3 stars, and manually curated data at 4 stars. All of the identified meta hub genes were found to be associated with all three diseases.

### 3.3 Meta hub genes-three disease mapping

**Fig. 11:**
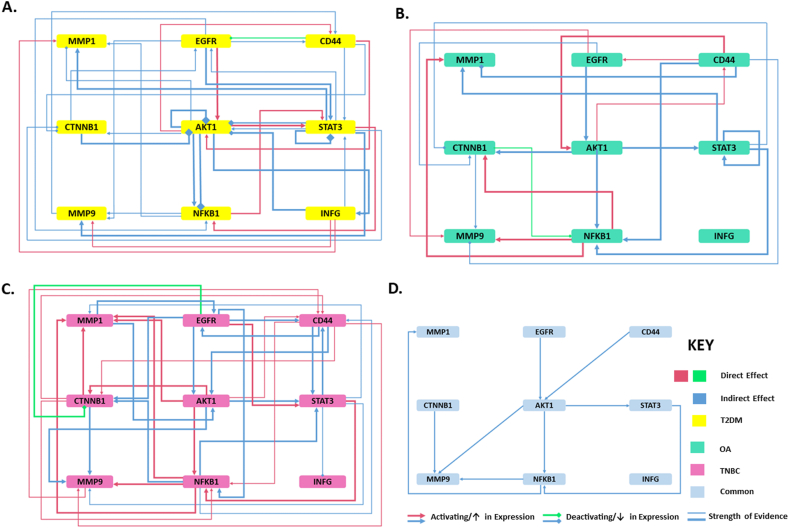
**Wiring diagram representing disease signalling networks.** Disease specific signalling maps are presented for **A-** Type 2 diabetes mellitus (T2DM) [36,37,[38], [39], [40], [41], [42], [43], [44], [45], [46], [47], [48], [49], [50], [51], [52], [53], [54], [55], [56], [57], [58], [59], [60], [61], [62]], **B-** Osteoarthritis (OA) [63,64,[65], [66], [67], [68], [69], [70], [71], [72], [73], [74], [75], [76], [77], [78], [79], [80], [81], [82], [83]], **C-** Triple negative breast cancer (TNBC) [84,85,86,87,88,[89], [90], [91], [92], [93], [94], [95], [96], [97], [98], [99], [100], [101], [102], [103], [104], [105], [106], [107], [108], [109], [110], [111], [112], [113], [114]], and **D-** 3 Disease mapping of T2DM-OA-TNBC signalling network.

The disease specific signalling maps were constructed from manually curated interactions data sourced by text mining and literature search. The disease specific signalling networks are depicted in Fig. 11A–C, whereas D shows a 3 disease (3-D) map derived from the superimposition and overlap between the disease specific interactions. This was done to identify signature meta-hub genes signalling mechanisms common to all three diseases and hence potentially characteristic of T2DM-OA-TNBC crosstalk.

In the 3-D map, *EGFR* and *CD44* were found to feed into *AKT1* activity such as the activation/supplementation of *STAT3,* *NFKB1,* *MMP9* and their downstream gene targets *MMP1* and *MMP9* (also activated/recruited by *CTNNB1*). At the core of this, *AKT1* can be seen to emerge as the hub of the meta hub genes; most interconnected and within signalling stream of all nodes except *CTNNB1* and *IFNG* *(*Fig. 11D*)*. Since the latter is not implicated in crosstalk with any other node, 8 candidate meta hub genes surfaced to be reigning the T2DM-OA-TNBC molecular crosstalk.

### 3.4 Discussion

This study employed a bio-computational approach to identify the molecular overlap between genes associating with T2DM, OA and TNBC using meta data of hub/core genes extracted from published literature. The interactions between various complex diseases paint a complex picture hence a focus was defined in the form of T2DM-OA-TNBC axes. The selection of these three morbidities as models of study is based on both disease burden and co-morbidity risk. While the cluster of complex diseases is extensive, diabetes, arthritis and cancer, prevalent worldwide and in Pakistan, are of particular interest. Since each of these diseases are heterogeneous, subtypes with greater incidence, elevated risk of co-morbidity and hence concurrently posing a greater health challenge were selected. The article selection strategy, as outlined in Fig. 1, involved the final inclusion of 252 research articles contributing 376, 347 and 537 hub/core genes associating with T2DM, OA and TNBC respectively.

These hub genes were imported and mapped onto FunRich to determine a molecular overlap between all three diseases. A signature of 31 hub genes were found to be common between all three datasets and hence was utilized for subsequent sequential analyses.

Recently published literature identifying hub genes specific to T2DM, OA and TNBC includes several articles not utilized here, since the scope of this study included articles matching the selection criteria up to 2023. Although these include the identification of disease specific key genes, however it is noteworthy that none of these hub genes were common to all three diseases, and therefore not relevant to this study design. Previously hubs genes common to T2DM and BC were identified, however since these did not match the BC subtype specificity criteria, these were not included in the analysis [31,115].

The 31 common signature hub genes were mapped with already reported/predicted genes associating with each of the three diseases, their hallmarks, and other characteristics, common factors, and pathways. Interestingly, all 31 genes were found to be present in GeneCards lists corresponding to T2DM and T2DM and 28 with OA. The 3 genes (*LCK,* *KRAS* *and* *UBC*) not present in the OA genes list from GeneCards were not omitted for subsequent analysis at this point to explore the possibility of identifying novel genes associating with OA and its co-morbidities. This analysis provided a glimpse into the clinic-pathophysiological niche of the interactome under study.

Genes associated with hallmarks for each of the three diseases were also extracted from GeneCards. All 31 genes associated with T2DM characteristics including insulin resistance, beta cell dysfunction and increased hepatic glucose output. For hyperglycaemic and inadequate insulin secretion phenotypes, 28 genes overlapped with the 31 hub gene signature. In case of hyperinsulinemia, an intersection corresponding to only 18 genes was determined. All 31 hub genes were shown to be implicated in joint pain, 30 genes each with chondrocyte degradation and synovial inflammation associated with OA, and 26 genes with asymmetric loss of cartilage space, consolidating their implication in OA pathogenesis. Furthermore, with the recent updates on hallmarks of cancer [116], overlap with all 14 hallmarks was assessed. A complete 31 signature overlap was seen with terms such as tumour promoting inflammation, sustaining proliferative signals, resisting cell death, angiogenesis, genome instability and mutation, deregulation of cellular metabolism and activating invasion and metabolism, indicating the role of these genes in tumorigenesis and cancer progression. These findings indicate at the functional determination of this interactome associating intricately with disease pathogenesis, particularly involving metabolic deregulatory mechanisms such as those associated with glucose level and output and particularly insulin resistance, a characteristic not only hallmark to T2DM, but also implicated in breast carcinogenesis and the development of OA [15,117]. Furthermore immune associating terms including chronic inflammation, particularly tumour promoting and synovium affecting inflammatory pathways were represented by the interactome under studying, highlighting the extent to which the inter-disease crosstalk is submerged within the interface of immunity and metabolism's intersection.

The molecular links between these three diseases may involve the integrative crosstalk of various cell signalling pathways translating into mechanisms and phenotypic hallmarks, also well represented by the these 31 meta hub/core genes. However a particular focus of this study is to elucidate on the immuno-metabolic crosstalk and disease initiation in existing morbid state characterized by low grade chronic inflammation and metabolic rewiring. This will provide comprehension on the extent of immuno-metabolic nature of the 3 diseases derived interactome, hence these terms are specifically highlighted amongst other findings.

Furthermore, common factors such as diet and lack of physical activity were studied and all 31 hub genes were shown to associate with these risk factors. Age is another risk factor affecting all three of these diseases, however it was omitted from the analysis. Over 25000 genes associated with it, according to GeneCards, and such a large dataset was unmanageable within the limits of the employed study pipeline.

Moreover, there are several disease characteristics and attributes common to all three diseases. Of particular interest, all 31 hub genes associated with oxidative stress, hypoxia and chronic inflammation. Metabolic reprogramming associated genes overlapped with 30 hub genes. Adipose tissue is reported to contribute significantly to the pathogenesis of not only T2DM, but also TNBC and OA. It is also a major contributor to breast tumour microenvironment and critically implicated in regulating OA progression [15,118]. All 31 hub genes overlapped with the adipose tissue genes, confirming their reported implication in adipose associated signalling and regulation of diseases. Since the focus of this study is to probe into the immuno-metabolic nature of association tying T2DM, OA and TNBC together, shared pathways including immune and metabolic pathways, were analysed next. All 31 hub genes were present in the genes listed for both these clustered pathways, again reflecting on the functional implication of the present interactome in immuno-metabolism.

It is also important to note, that here, the terms ‘metabolic pathways’ and ‘immune pathways’ are meta terms. These, each in itself includes not a single pathway but multiple signalling pathways and their crosstalk leading to interactive networks and consequently, devising highways implicated in metabolism and immune responses, respectively.

The functional enrichment for these 31 hub genes was also studied on EnrichR. It computes several types of enrichment scores such as *p* value derived from Fisher exact test, and adjusted *p* value by applying Benjamini-Hochberg method [119]. The most enriched terms of particular interest amongst GO BP category included apoptotic and proliferation regulation, and miRNA metabolic processes as shown in Fig. 6D. Additionally, cellular response terms in reference to reactive oxygen species, oxidative stress, *interleukin* *6* and *9*, lipid, cytokine stimuli, peptidoglycan, inflammatory response and its regulation, positive regulation of macromolecule metabolic process, nitrogen compound metabolic process, protein serine/threonine kinase activity, cellular metabolic process, *interleukin-1* *beta* production and intrinsic apoptotic signalling, cytokine mediated signalling, regulation of vitamin D biosynthetic process, *JAK-STAT* mediated receptor signalling pathway, *tumour* *necrosis* *factor* mediated signalling, regulation of cytokine production in inflammatory response and T-helper 17 cell differentiation amongst other significantly enriched processes not depicted in the top ten terms (Fig. 6A). MF terms such as insulin-like growth factor binding, tumour necrosis factor and chemokine binding activity, with RNA polymerase II binding were amongst significantly enriched terms. These again, highlight the over-representation of immuno-metabolic biological processes for the interactome.

Moreover, five different pathway analyses were performed to support a broader coverage and in-depth understanding of the significantly enriched pathways. According to KEGG database, some of the top enriched terms included pathways in cancer implicating 24 out of the 31 input genes, AGE-*RAGE* signalling (12 genes) and *PI3K-AKT* pathway (14). Furthermore, immune related terms including but not limited to T-h17 cell differentiation, IL-17, T cell and toll-like receptors signalling pathways, and cancer related terms such as breast cancer were also included. Osteoclast differentiation, focal adhesion, *HIF1* signalling, insulin resistance, adipocytokine signalling pathway, *NFKB* signalling, *insulin*, *mTOR,* *AMPK* and *p53* signalling pathways were amongst other significantly enriched terms. The results from other databases were found to be comparable with similar or functionally related pathways. Some other terms of interest from WikiPathways included cancer immunotherapy by PD-1 blockade, mammary gland development, endochondral ossification, folate metabolism, role of altered glycosylation of MUC1 in tumor microenvironment, factors and pathways affecting *insulin-like* *growth-Akt* signalling, miRNA involvement in the immune response in sepsis, regulatory circuits of *STAT* signalling and *leptin-insulin* overlap. Additionally, according to REACTOME, immune system and *interleukin* signalling, *NFKB* activation, cellular responses, metabolism of proteins, carbohydrates glycosaminoglycan, *NLRP3* inflammasome and other immune related pathways were also reported, with the term *interleukin* signalling being most significantly enriched (21 genes). Panther pathways also displayed enrichment for similar terms, particularly inflammation mediated chemokine and cytokine signalling, however returned the least number of enriched terms overall. Amongst the top significant terms from Elsevier pathways, proteins involved in altered expression in cancer metastases, breast cancer related terms, and specifically, *STAT3* and *NFKB* mediated activation of inflammation induced tumorigenesis were listed. It also included genes with mutations and proteins with altered expression both associated with cancer metabolic reprogramming, *IG1R/AKT* signalling in breast cancer, metabolism of thyroid hormones in adipose tissue, glutamine in cancer and overall *mTOR/p53* mediated cell metabolism. Diabetes associated activities such as adiponectin synthesis, adipokines by adipocytes, insulin signalling and resistance, and diabetic complications, were in line with other databases. As for OA, osteoclast and osteoblast related terms were also amongst the significantly enriched terms. Further probing into these pathways may provide in-depth understanding of the deeply rooted implication of the meta-hub genes in immuno-metabolic functional regime.

Moreover, the most number of genes associated with hub protein *ESR1* (17 genes) and for transcription factor, it was *SP1* (17 genes). Previously, genetic variant of *estrogen* *receptor* *1* *(ESR1)* has been associated with altered risk of TNBC [120], implicated in enhancing insulin sensitivity [121], and also associated with reversing osteoarthritic phenotype in OA chondrocyte [122]. The transcription factor Sp1 is reported to maintain a tissue specific mechanism for the regulation of target gene expression and is not only reported to mediate oncogenesis, but has also been studied in context to its role in OA and T2DM [123,124].

For miRNA, hsa-miR-155-5p was the most significantly enriched term, associating with 13 genes. This miRNA is reported to positively mediate glucose metabolism [125], and associate with diabetes and its complication such as diabetic neuropathy [126]. Interestingly, miR-155 has been termed the master regulator of inflammation [127], and miR-155-5p in particular has exhibited a protective role against OA [128]. Recently, miR-155 has also been reported as a diagnostic marker for TNBC [129], and for *ER* positive BC, it is shown to mediate metabolic rewiring stemming from cellular reprogramming in response to lack of estrogen [130]. In addition to this, it has been associated with T2DM-BC crosstalk [131], hence extending this axis to include OA, its multifaceted role in disease pathophysiology establishes another molecular link between T2DM, OA and TNBC.

Corresponding to another category, ClinVar presented about half of the terms to be associated with cancer, DisGeNET showed a diverse range of cancer terms including breast cancer triple negative neoplasms, osteosarcoma and other diseases including but not limited to diabetes mellitus, arthritis; specifically osteoarthritis, and other phenotypes such as inflammation, synovitis and obesity. Much fewer terms yet on similar themes were also presented by Jensen and OMIM diseases databases. Hallmark based significantly enriched terms of particular interest included EMT, *TNF-alpha* signalling via *NFKB*, inflammation response, hypoxia, glycolysis and signalling pathways mediated by *JAK-STAT3*, Interleukins, *AKT* *and* *p53*. This again reflects on the implication of the common hub genes’ signature in disease pathogenesis on the immuno-metabolic axis, supporting previously stated findings. Marker terms such as cancer stem cell and adipose derived-stromal and stem cells and metabolite Simvastatin were found to be significantly associated with our 31 hub genes signature. As previous discussed, adipose tissue provides a common ground between these three diseases and is of particular significance. To add to this, their association with cancer stem cell signifies their role in carcinogenesis and progression. Simvastatin is a lipid regulator drug, given to T2DM patients to treat dyslipidemia [132]. In OA, it is reported to induce a chondro-protective niche by reducing the expression of matrix metalloproteinases [133]. In case of TNBC, treatment with this drug induced ferroptosis in cancer cells in vitro [134].

Further on the therapeutics perspective, drug target terms were also assessed and one of the top enriched was Quercetin, implicating 8 hub genes including *AKT1,* *EGFR,* *MMP2* and *MMP9*, from amongst the meta hub genes. It is a naturally occurring bioactive compound, specifically a flavonol belonging to the group of flavonoids, with potent anti-oxidant and anti-inflammatory properties investigated for diabetic, arthritic and cancer models [135]. Hence, its multipurpose application in therapeutics establishes a consolidated ground for drug repurposing and targeting multi-morbidities on the T2DM-OA-TNBC axes, yet further research is required to validate this.

Specificity terms across sources included adipose tissue, whole blood, breast cancer cell line, immune system, synovial tissue, osteocyte, chrondocytes, and pancreatic progenitor cells. It also encompassed compartments such as NFKB and TORC1 complexes, in line with the tissues most affected by the diseases’ triad. Interestingly, these included sites and tissues most affected or crucially associated with the 3-Ds.

Next, a PPI network with 31 nodes was generated. MCODE analysis on Cytoscape identified one cluster existing within the network, with *IFNG* as its seed. This is achieved by implementing a graph theory based algorithm, that detects molecular complexes within large PPI networks, through identifying densely interconnected sub regions, or clusters within a locally dense node serving as seed and from which the cluster is derived [26]. Top 10 genes were retrieved as core genes within the module.

For meta hub gene identification, network topological properties-analysing algorithms were utilized and genes ranked accordingly to identify genes of greater significance within the network. The outcome of Degree and MCC methods gave identical rankings and, Density of Maximum Neighborhood Component (DMNC) the most diverse, as expected. A combined top 10 meta hub genes list was compiled based on the genes ranking higher by the greatest number of methods. There were 3 genes overlapping between the top 10 hub and core genes, each, hence giving rise to a total of 17 genes for subsequent analyses.

These overlapping genes which formed the sub interactome, were reanalysed for functional enrichment using Cytoscape plugin ClueGo, which performed the GO analyses for all three categories. Fig. 8A shows the most enriched BP terms labelled for distinct clusters based on functionally grouped terms (comprising of 68 over represented terms, each denoted by a node (based on a KappaScore group = 9), with functionally related groups also depicted. Their crosstalk included term-term interactions, with four larger clusters; particularly fibroblast proliferation (darker green), regulation of fibroblast proliferation (orange), response to cadmium ions (maroon) and positive regulation of miRNA metabolic processes (pink) are identified. Each colour represented a major cluster of terms signifying a functionally grouped sub network Furthermore, other enriched terms included positive regulation of vascular associated smooth muscle cell proliferation, cellular response to estradiol, regulation of carbohydrate catabolic process and post transcription gene silencing, NO synthetase regulator activity, extracellular matrix assembly, and release of cytochrome C from mitochondria. For the MF terms, four main groups were determined (12 representative terms and KappaScore groups = 4), with interactions within the NO synthase regulator activity, and core promoter sequence-specific DNA binding associated nodes. For CC, only two terms were found: transcription repressor complex and euchromatin (KappaScore groups = 2).

For KEGG analysis, there were 52 enriched terms, with KappaScore group of 1 and the most over-represented term being proteoglycans in cancer, as shown in bold (Fig. 8D). These analyses conclude the implication of the sub interactome in cell proliferation, particularly of fibroblasts, cellular responses, metabolic processes, cancer pathways, and specifically, the role of proteoglycans in cancer, representing the singular main functional group emerging from the pathway analysis. Additionally, immune associated terms such as *TNF,* *IL17,* B cell, T cell and Th1 signalling pathways were also enriched, This again validates in in silico, the potential role of the T2DM-OA-TNBC interactome in disease initiation, and carcinogenesis in particular, converging on metabolic, immune and cancer associated pathways.

However, it is important to note that the terms the hub genes are enriched for does not necessarily equate to a positive association with the particular function. It is crucial to bring into context the expression of each of these genes to comprehend the nature of their role in that particular phenotype. The expression based validation of the 17 meta-hub genes was executed using a combined disease specific databases- and literature search based approach. Genes exhibiting a similar expression pattern, particularly those up-regulated across all three diseases, were of particular significance. These included *MMP9* (through databases), and others (*MMPs,* *NFKB1* and *CD44*) based on manually curated information on expression reported in previously published articles.

To identify the molecular drivers of this sub interactome, further shortlisting was done based on several parameters dictating prioritization as shown in Fig. 10.

Confirmation of the gene-disease association for the prioritized meta hub genes was done on Diseases database. It assigns a z score based on the gene's association with disease taking into account the co-occurrence of gene and disease terms in text by chance and through an actual association. These scores are then converted into confidence measured by stars. All 9 meta hub genes were found to be associated with 3-Ds, with 3 star and above confidence level. This marked the completion of the bio-computational analysis framework, generating an output of 9 candidate meta hub genes potentially associated with T2DM-OA-TNBC axes.

Probing into the gene-gene interactions within this sub interactome identified for T2DM-OA-TNBC by devising a signalling circuit may provide further valuable insights into the switch mechanisms employed by diseased cells and tissues to accommodate a secondary pathogenic cascade of signalling dysregulations underlying the emergence of multi-morbid state. While these meta hubs may be critically implicated in disease driving signalling, the decision making ability at molecular level is shared by a set of switch genes. These genes exercise their regulatory control over the cell through the feedback mechanism ubiquitously embedded within the cellular systems [136]. Hence, it is critical to study potential switch genes within this sub interactome and to further elucidate on the secondary regulatory players. For this, the 9 candidate meta hub genes were studied for their regulatory patterns within the system to manually curate gene-gene interactions data. Regulatory networks representing disease specific signalling maps for all diseased states were constructed and superimposed to bring to surface the regulome perpetuating T2DM-OA-TNBC axes.

Finally, these genes were screened for their potential as central transducers of signalling within the T2DM-OA-TNBC interactome. Disease specific regulatory networks were superimposed to identify common molecular routes that could drive the switch mechanisms underlying inter-disease crosstalk and initiation of disease, particularly in case of co/multi-morbidity.

Amongst these, 9 meta hub genes, all except *IFNG*, were implicated in T2DM-OA-TNBC overlapping crosstalk. *Interferon* *gamma* *(IFNG*), is a pro inflammatory cytokine, reported to modulate and increase the plasticity within the immunopeptidome in TNBC, shown in a study conducting *IFNG* treatment of TNBC cell line MDA-MB-231, leading to diversified antigen processing presentation [137]. This could potentiate personalized medicine based cancer vaccine strategies in the near future. It was identified as the seed node within the clustered network. Ironically though, after bringing into context disease specific signalling pathways and common molecular routes signifying core regulatory nodes within 3-D crosstalk, it was found to not interact with other nodes within the regulatory network underlying T2DM-OA-TNBC associations. Hence, while pursuing *IFNG* as a candidate meta hub gene may be promising, supported by its interconnectivity on T2DM and TNBC specific signalling maps, its implication with context to other candidate nodes was not seen on the 3-D map, therefore it will not be discussed further. Hence 8 meta hub genes depicted potential molecular interplay on the 3-D signalling map underlying T2DM-OA-TNBC axes. These included *AKT1,* *NFKB1,* *CTNNB1,* *EGFR,* *MMP1,* *MMP9,* *CD44*, and *STAT3*.

*Akstraintransforming(AKT)*, is a critical regulator of the *PI3K-AKT* signalling pathway. This pathway is crucially implicated in core cellular mechanisms such as survival, metabolism and autophagy, and activated in response to multiple stimuli including but not limited to nutrients, growth factors, cytokines and hormones [138]. It is a serine/threonine kinase which exists in three isoforms. *AKT1* is involved in glucose metabolism along with its more predominantly implicated isoform A*KT2*. *AKT2* also specifically regulates fibroblast differentiation, particularly adipogenesis [139], and mediates pancreatic beta cell response to endoplasmic reticulum stress [140,141].While *AKT1* is also shown to be crucial for fibroblast proliferation and other cellular processes [142]. This is supportive of present study's findings on enriched fibroblast proliferation and its regulatory pathways. Previously, the potential of combinatory inhibition of glycolysis and glutaminolysis in fibroblasts to address metabolic reprogramming in another type of arthritis; rheumatoid arthritis has been studied [143], reflecting on the critical role of metabolic rewiring within fibroblasts and its effect on disease development. Furthermore, *AKT1* is also reported to inhibit breast cancer cell migration, by regulating EMT proteins [144] promote local tumour growth [145], and critically implicated in proliferation in TNBC. It is important to note however, that its expression in TNBC is relatively lower in comparison to other BC subtypes. With reference to its role in OA, its expression in chondrocytes has been implicated in the negative regulation of calcified cartilage formation [146].

*Nuclearfactorkappabeta(NFKB)*, a transcription factor, is not only a critical regulator of inflammatory pathways, but its subunit Rel, crucially modulates metabolism in B cells [147]. Moreover, in cancer, *NFKB* is reported to mediate cellular response to nutrient starvation leading to metabolic adaption. *NFKB* mediated Inflammasome activation in response to metabolic imbalances is also reported. In breast cancer in particular, its expression is associated with 10 fold chemoresistance [148]. Moreover, it is associated with inflammatory biomarkers and catabolism up-regulation and anabolism down-regulation in chondrocytes, contributing to OA [149]. In T2DM, it is activated in response to hyperglycemia and is associated with vascular complications [150].

The gene encoding beta-catenin, *CTNNB1,* is implicated in energy homeostasis and its underlying glucose metabolism [151]. It is a crucial player of the *WNT* signalling and is known to interact with *transcription* *factor* *7* *like* *2* *(TCF7L2),* *Forkhead* *box* *protein* *O* *(FOXO),* and *HIF1A,* and previously implicated in the diabetes-cancer link [31,152]. In OA, its dysfunction is a critical contributor to adiposity, chronic inflammation and diet induced insulin resistance in skeletal muscles [153].

*Epidermalgrowthfactorreceptor(EGFR)* is overexpressed (constitutively) in up to 50 % of TNBC cases and is found to correlate with *CTNNB1* expression in TNBC tissue [154,84]. It is reported to phosphorylate beta catenin, leading to EMT, while also reduce membrane associated beta catenin. It has been implicated in the diabetes-associated vascular complications, particularly kidney dysfunction [36]. Whereas in case of OA, it has depicted a protective niche by maintaining cartilage homeostasis and increasing joint lubrication, hence negatively regulating disease progression [155,156].

*Matrixmetalloproteinase1(MMP1)* is a collagenase, induced by *AKT1* signalling, and crucially involved in the ECM degradation [157]. In OA, it is particularly involved in mediating the joint destruction by allowing for articular cartilage breakdown. Its expression is reported to be high in T2DM patients and is associated with a role in diabetic complications; wound healing in particular [158,159]. It is also reported to be overexpressed in TNBC, and associated with growth apoptosis, and metastasis [85].

Another member of the matrix metalloproteinases family, *MMP9* is a gelatinase, implicated in adipose tissue signalling and its dysregulation, highlighting its critical role in adipose homeostasis and higher plasticity in responding to nutrients [160]. Furthermore, *MMP9*'s involvement in regulation of metaflammation associated with obesity [160], signifies another immuno-metabolic link at molecular level.

*Clusterofdifferentiation44(CD44)* is a transmembrane glycoprotein that acts as homing cell adhesion molecule (HCAM) and principally receptive to Hyaluronan, an extracellular matrix glycosaminoglycan [161]. *CD44*'s role in adipose tissue inflammation, particularly diet induced, and its association with insulin resistance and T2DM has been established previously [[162], [163], [164]]. Furthermore, it has been reported as a marker for breast cancer initiation [86], particularly for TNBC [87,165]. It is sensitive to changes within microenvironment and this may contribute towards its role in cancer initiation [166]. It is also associated with increased cytokine mediated synoviocyte proliferation in OA [167].

*Signaltransducerandactivatoroftranscription3(STAT3)* mediates insulin resistance in skeletal muscle, and mitochondrial gene expression and electron transport chain mechanisms in pancreatic beta cells [168,169]. It is reportedly overexpressed and constitutively active in TNBC, exhibiting oncogenic potential by inhibiting apoptosis and promoting cell survival and growth [88]. Furthermore it is also associated with OA progression through the mediation of *NFKB* pathway, contributing to joint destruction [63].

At the crux of these interacting nodes, *AKT1* is a critical meta hub gene, exercising its role as the central transducer of signals, hence, nodes directly upstream and downstream to it may be further implicated in the interactome's functional centrality. Hence, a molecular concoction of 6 critical nodes achieved through the subsequent molecular equation, may potentially dictate the molecular reprogramming underlying the immuno-metabolic origin of disease.

EGFR,CD44=>AKT1=>NFKB1+STAT3+MMP9

Probing into the crosstalk of these 6 nodes on immuno-metabolic interface of disease causing mechanisms, may provide a redirection to understanding the underlying characteristics common to all three diseases, within the context of this regulatory axis. For instance understanding the interplay of metabolic switches, other drivers of metabolism and chronic low grade inflammation, a feature prevalent in many diseases including metabolic disorders, further complicated with the development of insulin resistance, oxidative stress and hormonal fluctuations may bridge the knowledge gap on the exact molecular mechanisms underlying T2DM-OA-TNBC association (s).

Considering the status quo of existing research on this, several molecular links already arise. The phenotypic characterization of severe insulin resistance in T2DM is associated with an atypical blood inflammatory response [170]. Insulin resistance is a causative mechanism, particularly for obesity associated OA [171] and cancer, hence it serves as a molecular tie between diabetes, OA and cancer. Moreover, latest trends in research focus on immune cell profiling to predict disease susceptibility and to correlate with disease pathogenesis, highlighting the significance of probing into the immuno-metabolic axis for metabolic disorders such as diabetes, along with OA and BC.

In line with this, inflammatory mechanisms formulate a well-established link between diabetes and other metabolic conditions including obesity, and metabolic syndrome [172]. Interestingly, these molecular connections extend to other morbidities with underlying metabolic deregulations at play, including but not limited to cancer and arthritis. Breast cancer is known to exhibit elevated glucose consumption and production of lactate, which contributes towards suppressing body's antitumor response [[173], [174], [175]]. Dysregulations within the metabolic pathways are critically implicated in tumorigenesis and its further development, hence focusing on targeting metabolic pathways may provide a promising avenue to block tumour initiation [176]. Furthermore, within the tumour ecosystem, immune cells play a significantly critical role, intertwined with metabolic reprogramming, and exhibit plasticity [177]. In parallel, metabolic programs within the immune cells dictate their activation and fate [21]. Pivotal to this crosstalk between metabolic rewiring and inflammatory pathway activations, are the *NFKB* and *AKT* pathways, mediating the immuno-metabolic centred crosstalk, potentiating transition in disease states.

Yet, it is challenging to pursue genes with pleiotropic effects as potential biomarkers. Hub genes within a network are the most interconnected of nodes, hence indicating at their centrality within the functioning and regulatory systems. Targeting *AKT* and *NFKB* and their pathways has been pursued as therapeutic strategies for T2DM [150,178], TNBC [179,180], and OA [181,182]. Another strategy to address this complexity is to identify the signalling axes based on the meta hub genes identified. Probing within the clustered networks of these meta-hub genes may then help identify downstream genes that can disconnect disease implicating feedback loops within signalling paths crucial for cell survival. For instance, *MMPs* may also be considered as potentially promising genes to target on the T2DM-OA-TNBC axes. While *MMP1* is reportedly elevated in TNBC, indicating at its potential diagnostic relevance, its inhibition is shown to inhibit malignancy in vitro, and *MMP9* has also been found on the therapeutic axis [85,183]. *MMPs* have also depicted potential for therapeutic interventions targeting T2DM complications associated with wound healing [184], and OA [185].

Furthermore, other meta hub genes identified also associate with clinical significance. For instance, *CD44*, implicated in insulin resistance, has been studied as a therapeutic target with potential for T2DM [186]. Research has also shown, targeting it may prevent immune triggered cytokine activation and development of chronic joint inflammation, ameliorating OA conditions [64]. Furthermore, it is already under study as a therapeutic strategy against TNBC, and identified as a cancer stem cell marker [165,187]. Similarly, *EGFR* holds therapeutic significance in case of diabetic complication leading to Kidney disease [188], for OA [156], and TNBC [189]. In case of *STAT3*, several of its inhibitors are under preclinical study for TNBC, and it is associated with overall poor survival [190]. It is also pursued for Metformin associated T2DM [37], and OA [191]. Targeting of *CTNNB1* too has exhibited therapeutic potential for OA [192], and TNBC [154]. Another study using anti-diabetic drug pioglitazone in Glioblastoma Multiforme lowered beta-catenin level [193], creating potential ground for T2DM therapeutic intervention studies targeting *CTNNB1*.

At the next level, reflecting on gene-gene interactions may lead us to identifying early disease driving molecular events which may involve the integration of signals from various inputs to orchestrate regulatory networks central to comprehending the origin of complex diseases. In line with this, understanding of disease associated gene sets may provide valuable insight into the co-localization of two or more diseases wired by an overlapping of their gene networking systems. This may in turn potentiate disease module discovery and perhaps even open avenues for targeting the molecular origin of disease initiation.

However, large gene networks underlying complex diseases and their signalling crosstalk pose a challenge for deriving insights with the system as a whole, hence these are subjected to network analysis such as module identification so that functionally grouped units or pathways involving genes implicated in the same biological functions can be recognized within networks of high modularity, i.e dense regions within the networks because of highly interconnected nodes, as compared to others [194]. Within this context, the identification of switch genes responsible for transition from one diseased state to another or comorbid condition, may facilitate comprehension of disease-disease relationships [195]. Interestingly, it has been observed that switch genes may be closer to each other in comparison to other nodes within gene regulatory networks underpinning specific diseases. This may be exploited further by studying disease-disease commonalities such as their molecular overlap in the shape of interactomes, such as the derivation of molecular equation identified in the context of T2DM-OA-TNBC crosstalk, through this study.

This has been made possible with the evolution within the field of medicine, particularly personalized medicine, leading to advances in technology and the generation of high throughput omics data which introduced another spin off trajectory in the name of network medicine [195]. A single disease is treated as a collection of patho-phenotypes associated with a set of mapped genes depicted not only as an interplay with other factors, but also exhibiting a molecular overlap and shared mechanisms resulting from an intricately executed crosstalk in cellular context. As if this was not complicated enough at disease level, further complexities arise in the face of disease-disease associations established through the expression of switch genes at molecular level, which exercise their regulatory reigns at cellular and higher levels. Unravelling the multi-layered associations between complex diseases at gene-gene interaction level, may potentiate biomarker discovery coupled with therapeutic interventions for drug repurposing and designing. In summary, pleiotropic gene modules such as the meta hub genes identified, sketching complex regulatory landscapes and exhibiting immuno-metabolic plasticity could be studied. Specifically, finding the molecular signature which leads to the interplay between multiple traits and phenotypes common to various complex diseases may elucidate on the molecular mechanisms underlying disease-disease associations [196]. In the context with T2DM-OA-TNBC inter-relationships, this may allow for comprehension of molecular bases of cancer initiation in diabetic/osteoarthritic models on immuno-metabolic axis.

While this study elucidates on the immuno-metabolic landscape of inter-disease relationships; potentially driven by an interplay between *AKT1* and *NFKB1* pathways, these findings may just be the tip of the iceberg. The origination of deregulatory perturbations within the genetic makeup may involve multitude of switch genes in action to sponsor pathogenic changes resulting in diseased outcomes. Hence further in silico and in vitro studies are warranted to dive into the depth of this crosstalk. Furthermore, limitations in the form of existing biases associated with resources utilized apply, and hence effort was made to rely on adjustments for multiple tests correction where possible. The three diseases under study served as models for this preliminary in silico research based on their inter-disease associations and shared molecular overlap. While the T2DM-BC association has been reported, and its underlying molecular mechanisms hypothesized, the OA-BC association is still unexplored and hence requires further scientific investigation. It is also interesting to note that bone is that most frequent site of metastasis for BC [197], and OA and BC both involve bone remodelling [198,199], further igniting interest in understanding the underlying molecular overlap.

The potential of this study lies in the application of its findings in understanding disease patho-physiologies of co-morbid patients, so as to potentiate appropriate therapeutic targeting and also contribute towards lowering the risk of co-morbidity in already diseased patients. Identifying and targeting meta-hub genes may potentially be the right track in addressing this public health concern. Additionally this may also enable drug repurposing to allow for already available drugs to be studied for and utilized for these inter-disease associations.

However, further extensive and thorough pursuit, involving other complex and chronic diseases with overlapping immuno-metabolic features is required to decode the origin of immuno-metabolic diseases. Moreover, each of these diseases under study are heterogeneous in nature and further categorized, with T2DM associating with varying complications, OA defined on the basis of affected area and TNBC subtyped into further classes, hence further stratification of diseases is required for clarity, in the future on the road to precision medicine.

## 4 Conclusion

Understanding complex polygenic diseases with the application of network biology concepts map the complexities within a functioning system into protein-protein interactions underlying healthy and diseased states. This enables the emergence of hub genes within the biological networks, highlighting the significance of their role within the biological system [200].

This study employed a systems biology approach to probe into the hub genes associating on the type 2 diabetes mellitus (T2DM)- osteoarthritis (OA)- triple negative breast cancer (TNBC) axes. In summary, the T2DM, OA and TNBC interactome comprised of 31 meta hub/core genes, with 27 functionally related genes that led to interlinked pathways and signalling networks underlying multi-morbidity. Perturbations within this interactome, particularly loss or gain of function mutations within these hub genes would essentially initiate a ripple effect of disturbance leading to a spectra of diseased phenotypic outcomes and associations. Understanding the global dynamics within such regulatory networks may provide insight into the disease causing-switch genes and their impact on the genetic circuits/diseasome underlying disease pathogenesis.

Ultimately, 8 meta hub genes constituting the molecular activation cocktail were found to potentially govern disease initiation and progression, particular implicating the immuno-metabolic axis, at the heart of this crosstalk. Interestingly, all these identified meta hub genes are clinically significant targets for addressing T2DM, OA, and TNBC, hence potentiating their relevance in cases of co-morbidities. In particular, the *AKT1-NFKB1* axis highlights the crux of immuno-metabolic crosstalk and can be probed further into to gain molecular insights into the immuno-metabolic origin of complex and chronic diseases such as T2DM, OA and TNBC, under study. Deciphering the molecular basis for the intertwined metabolic and immune states may potentiate in-depth understanding of the molecular driver events leading to such immuno-metabolic diseases, particularly the discovery of biomarkers for identifying the immuno-metabolic origin of TNBC in diseased patients, in part addressing the incidence of multi-morbidities which are a prevalent health concern worldwide.

## Role of funding source

Not applicable.

## CRediT authorship contribution statement

**Ilhaam Ayaz Durrani:** Conceptualization, Data curation, Formal analysis, Investigation, Methodology, Resources, Software, Validation, Visualization, Writing – original draft, Writing – review & editing. **Peter John:** Conceptualization, Data curation, Formal analysis, Investigation, Methodology, Project administration, Resources, Software, Supervision, Validation, Visualization, Writing – original draft, Writing – review & editing. **Attya Bhatti:** Conceptualization, Data curation, Formal analysis, Investigation, Methodology, Project administration, Resources, Software, Supervision, Validation, Visualization, Writing – original draft, Writing – review & editing. **Jahangir Sarwar Khan:** Resources.

## Declaration of competing interest

None Declared.

## Abbreviations

***RAC1***: ras related C3 botulinum toxin substrate 1
***PAK1***: p21 (RAC1) activated kinase 1
***HSP90AA1***: heat shock protein 90 alpha family class A member 1
***FOS***: fos proto-oncogene
***TP52***: tumour protein 52
***JUN***: jun proto-oncogene
***miR***: microRNA
***TNF***: tumor necrosis factor
***BCL-2***: B-cell lymphoma 2
***TP53***: tumour protein 53
***IL6***: interleukin 6
***AGE***: advanced glycation end products
***RAGE***: receptor of AGEs
***HIF1(A)***: hypoxia inducible factor 1 (alpha)
***KRAS***: Kristine murine sarcoma virus2 homolog
***JAK***: janus kinase
***PI3K***: phosphoinositide 3-kinase
***mTOR***: mammalian target of rapamycin
***MUC1***: mucin 1
***NLRP3***: nucleotide binding domain, leucine-rich-containing family, pyrin domain-containing 3
***IG1R***: insulin like growth factor 1
***MMP2***: matrix metalloproteinase 2
