# Supplementary material for: Network medicine based approach for identifying the type 2 diabetes, osteoarthritis and triple negative breast cancer interactome: Finding the hub of hub genes
Source: Heliyon. 2024 Aug 22;10(17):e36650. doi: 10.1016/j.heliyon.2024.e36650 (PMC11401126; doi:10.1016/j.heliyon.2024.e36650)
Supplement: Multimedia component 1 [file mmc1.docx]

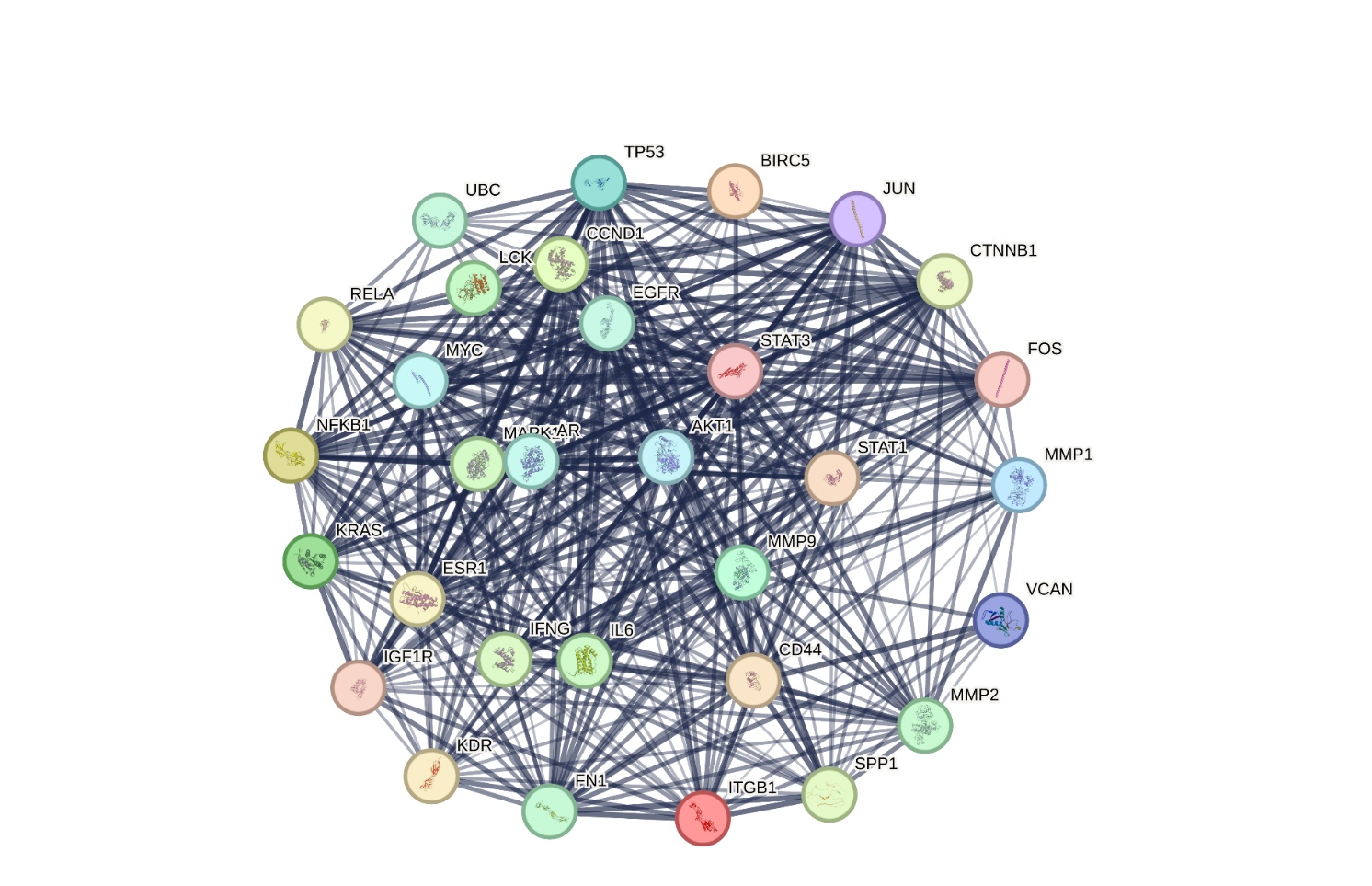


**Supplementary Figure 1 Protein-protein interaction (PPI) network for common hub genes.** The nodes (31 in number) are represented by spheres and the edges connecting two nodes depict interactions.

**Supplementary Table 1** MCODE cluster top ten genes. Analysis based scores are represented for each of the genes. The seed of the cluster was identified as IFNG.

| **Sr. No.** | **Top Ten Genes** | **MCODE Score** | **Cluster** |
| --- | --- | --- | --- |
| 1 | *IFNG* | 19.605 | Seed |
| 2 | *FOS* | 19.526 | Clustered |
| 3 | *MMP2* | 19.420 | Clustered |
| 4 | *CD44* | 19.420 | Clustered |
| 5 | *KRAS* | 19.348 | Clustered |
| 6 | *MMP1* | 18.910 | Clustered |
| 7 | *AKT1* | 18.892 | Clustered |
| 8 | *NFKB1* | 18.892 | Clustered |
| 9 | *CCND1* | 18.892 | Clustered |
| 10 | *STAT3* | 18.892 | Clustered |

**Supplementary Table 2 Hub gene identification.** Ranking of hubs and their corresponding networks are depicted according to Cytohubba plugin's algorithms. Abbreviations: MNC- maximum neighbourhood component; MCC- maximal clique centrality; DMNC- density of MNC; EPC- edge percolated component and CC- clustering coefficient.

| **Method** | ***Degree*** | ***MNC*** | ***MCC*** |
| --- | --- | --- | --- |
| **Rank** | 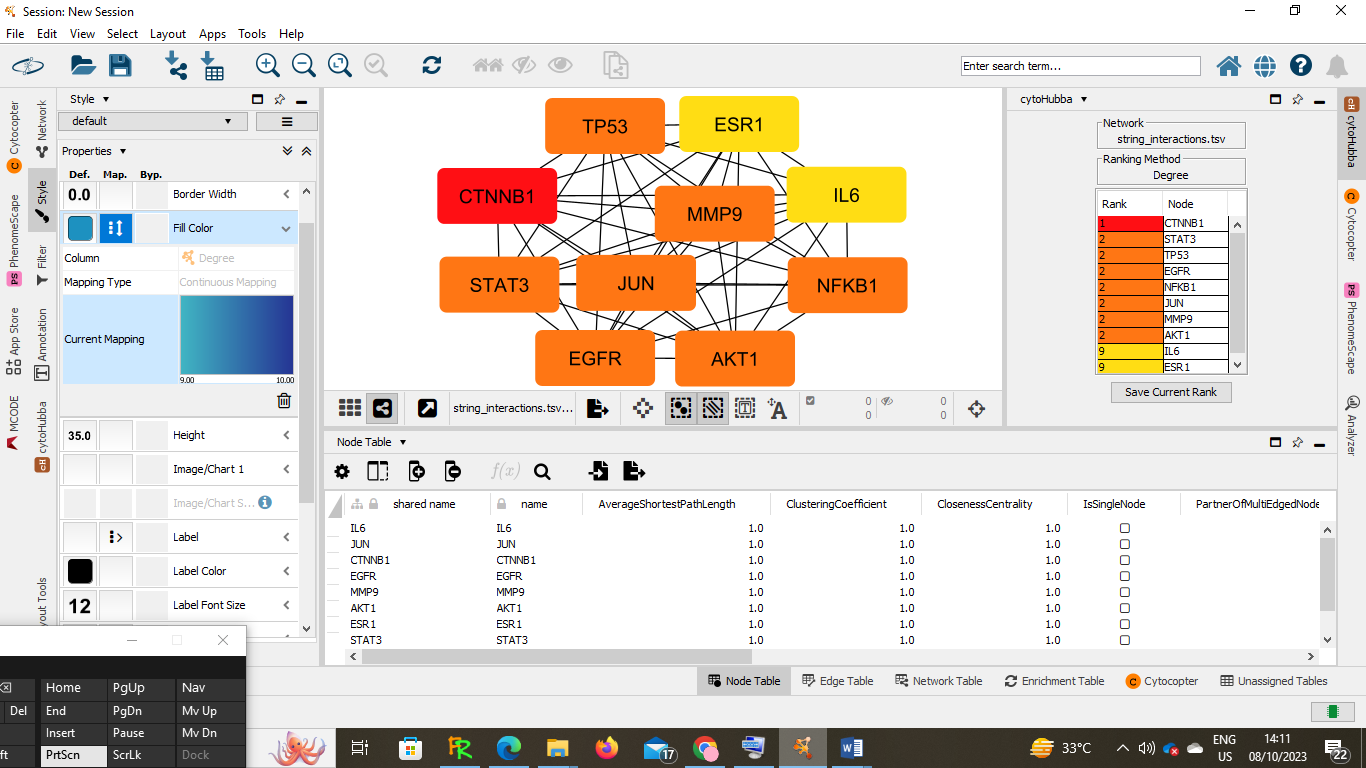 | 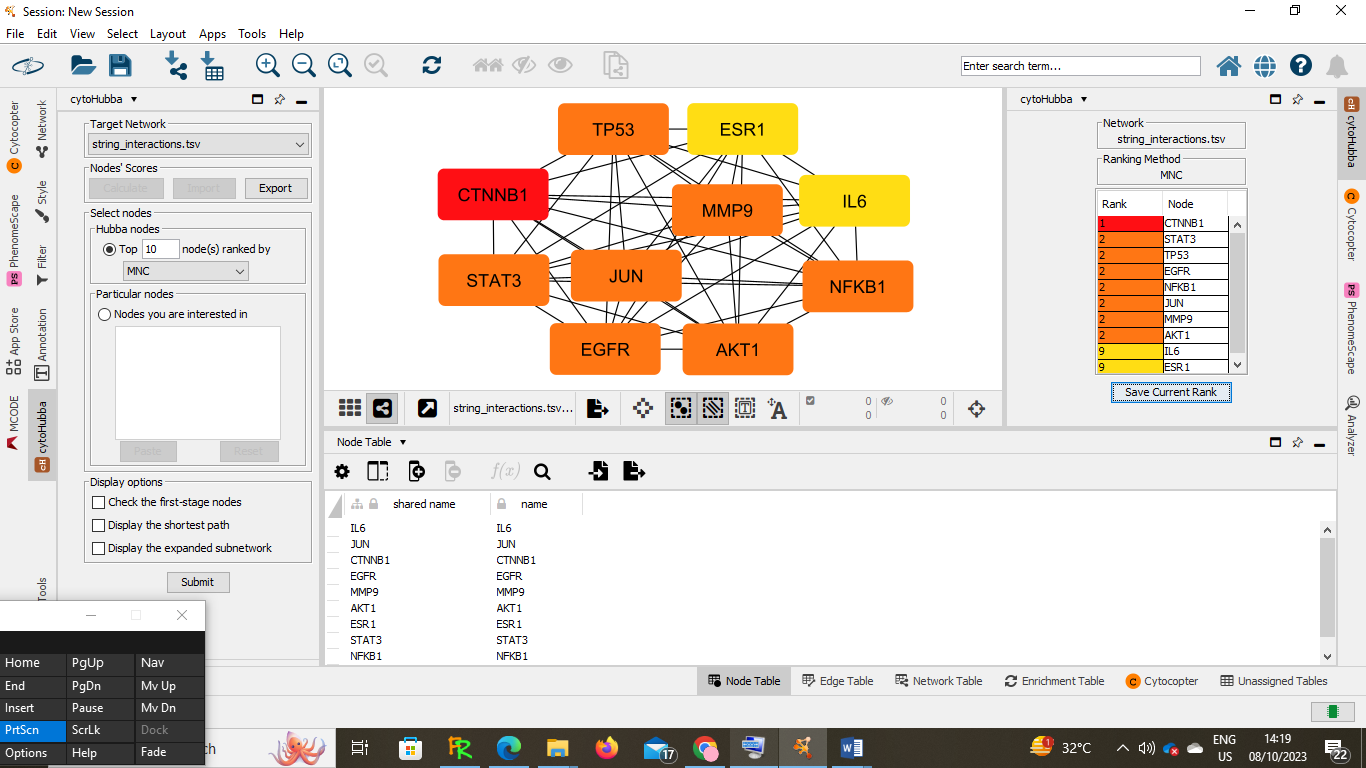 | 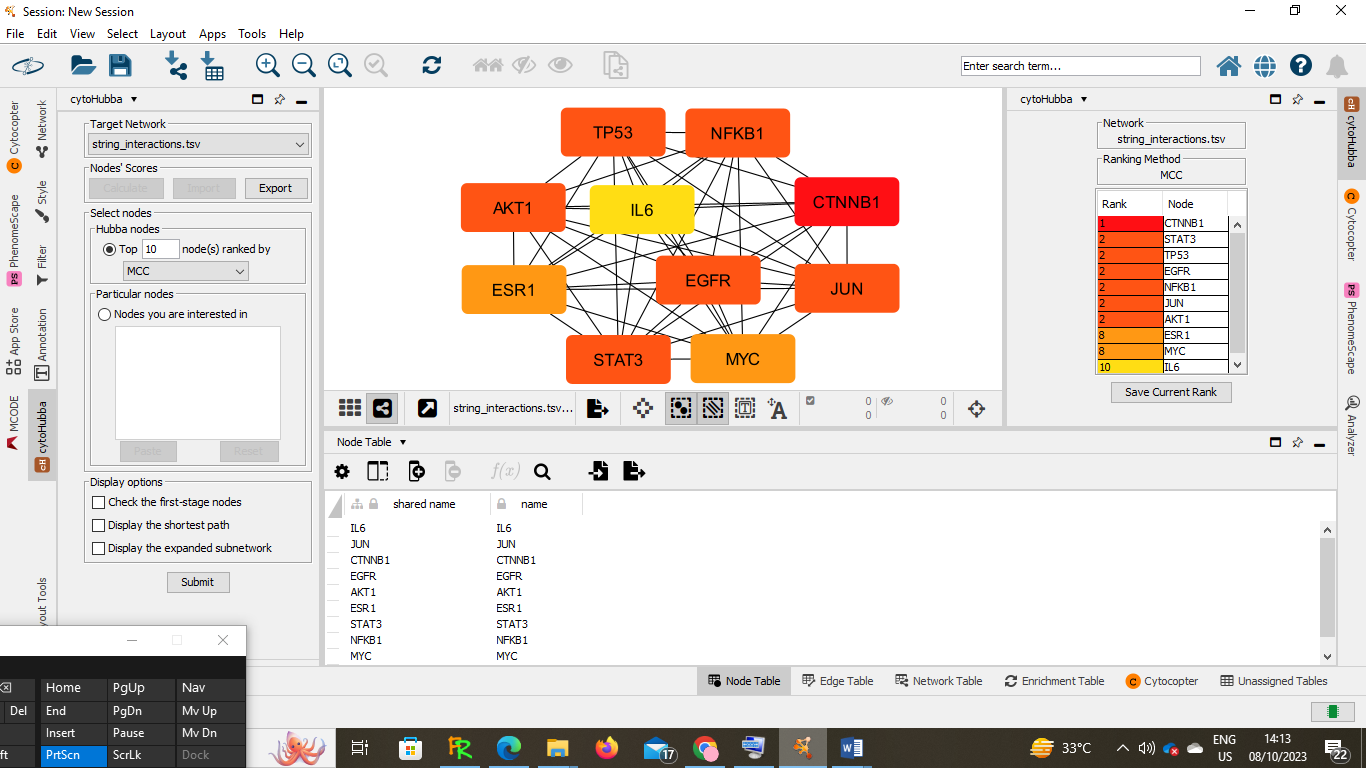 |
| **Network** | 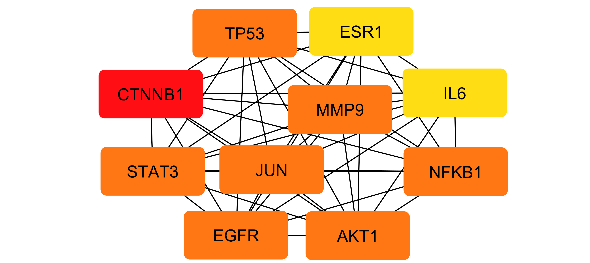 | 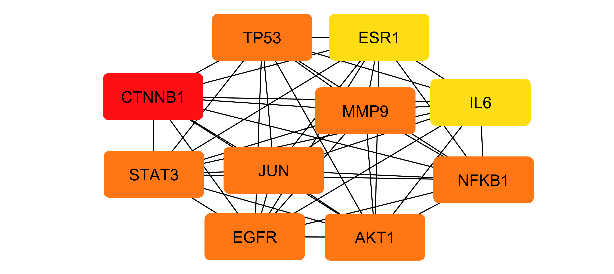 | 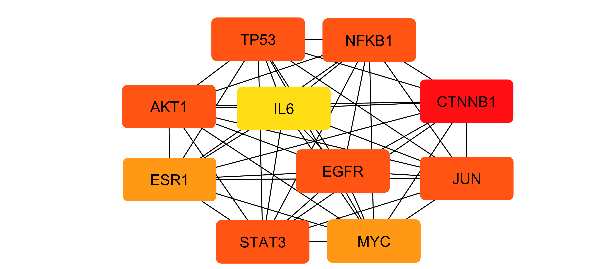 |
| **Method** | ***Betweeness*** | ***Closeness*** | ***Radiality*** |
| **Rank** | 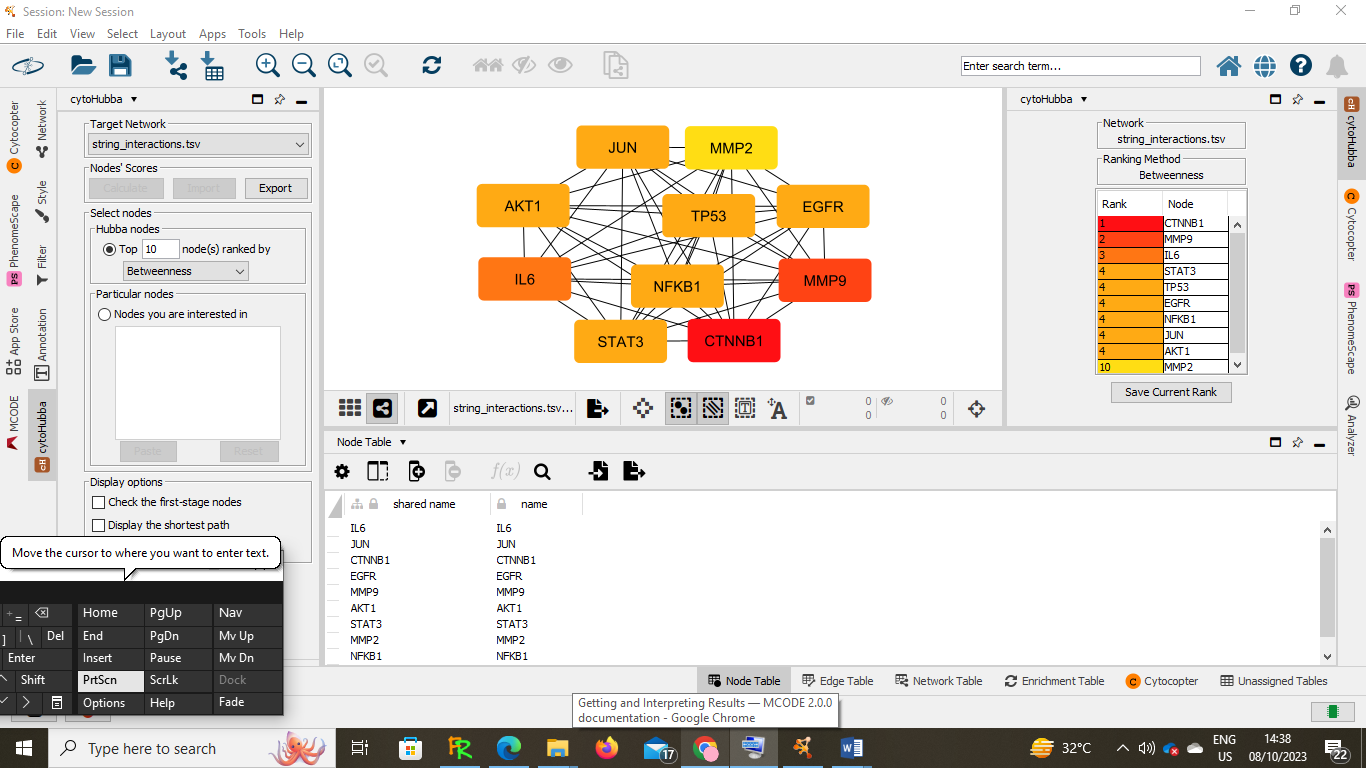 | 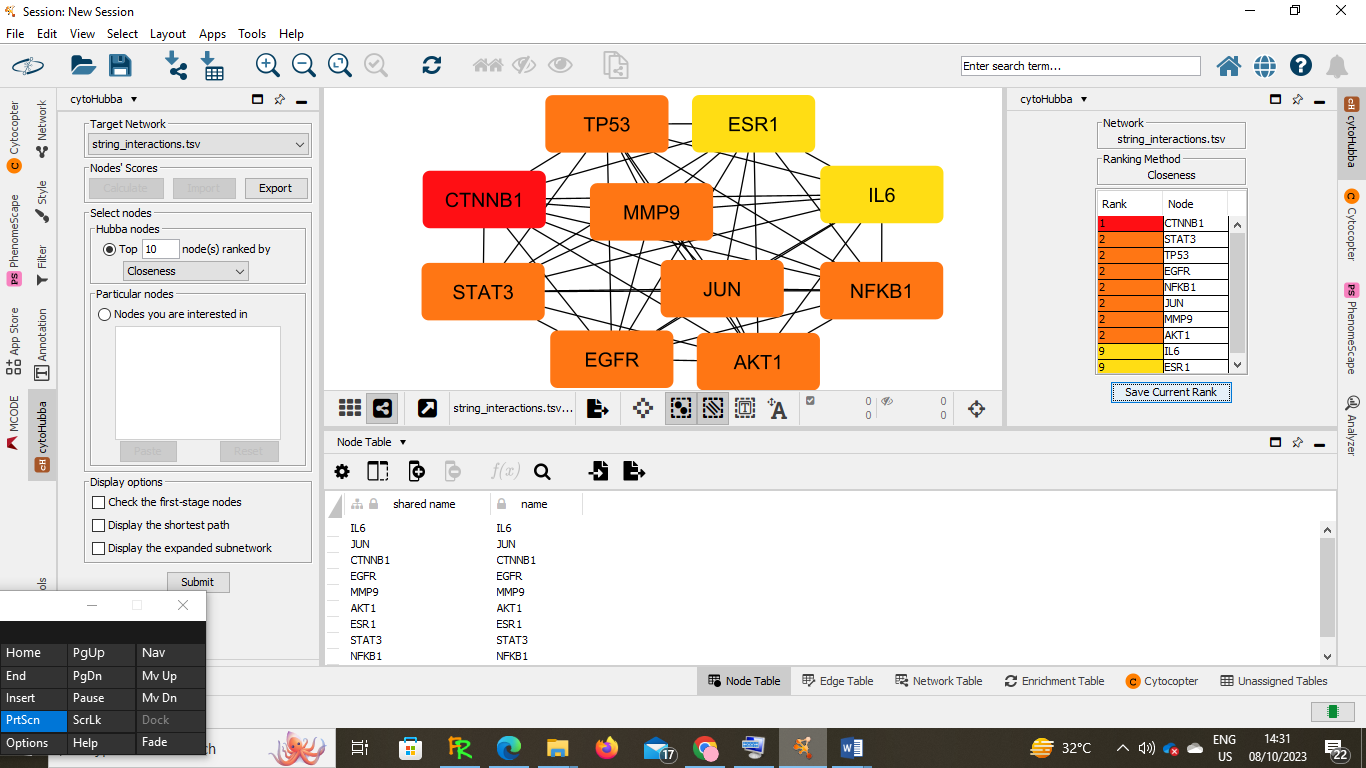 | 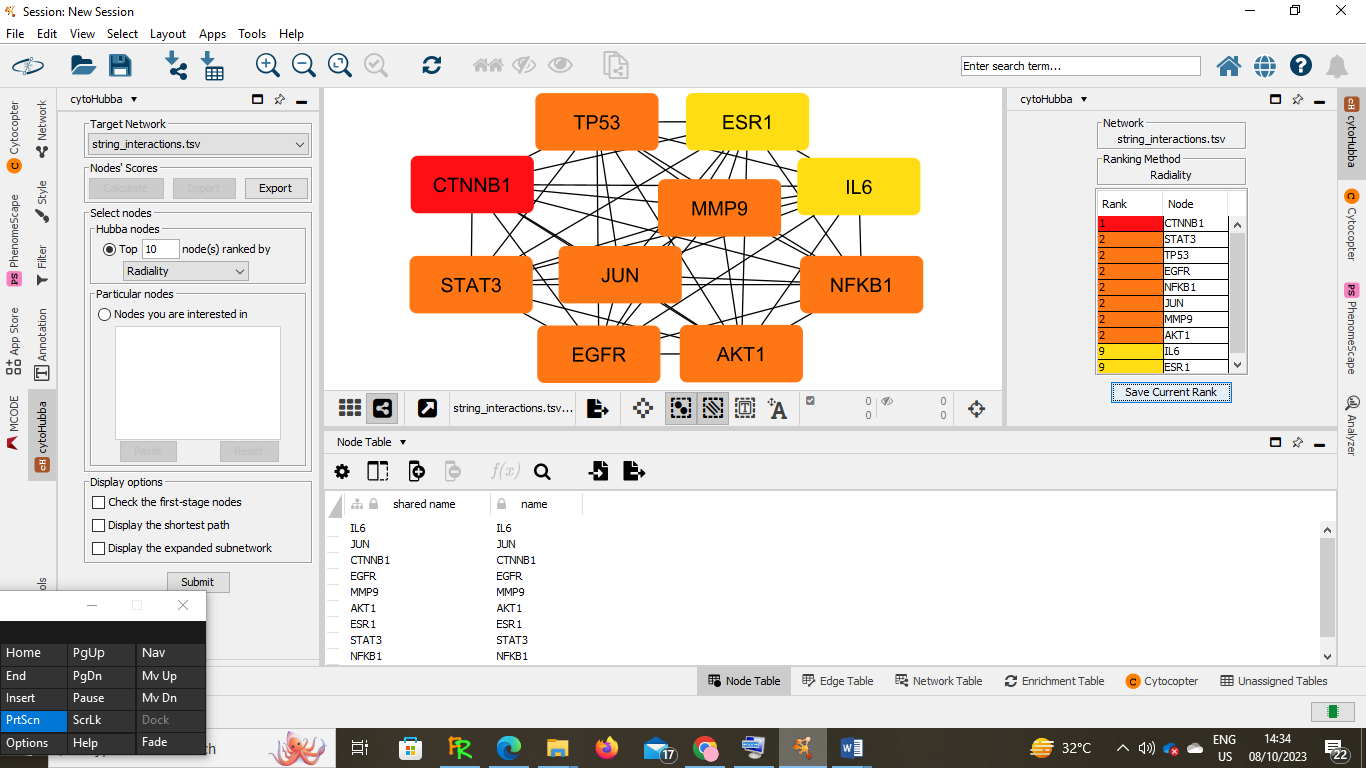 |
| **Network** | 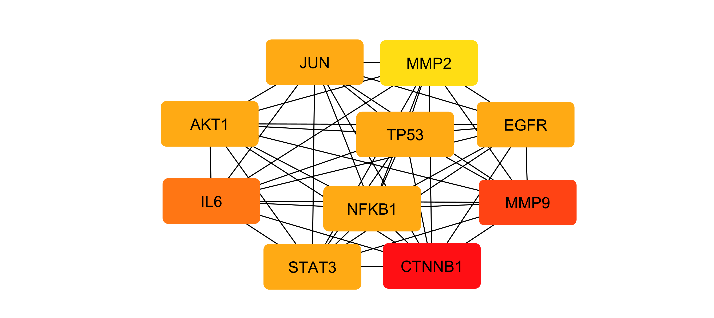 | 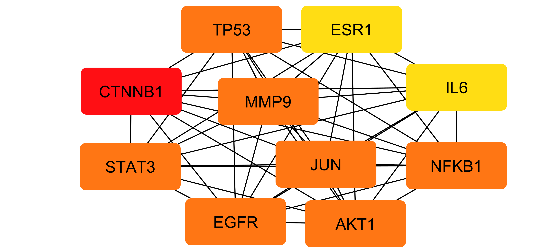 | 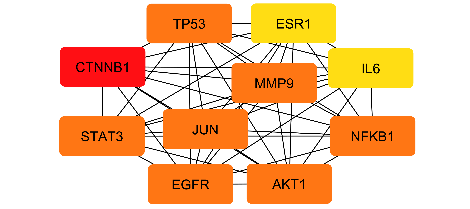 |
| **Method** | ***EcCentricity*** | ***DMNC*** | ***BottleNeck*** |
| **Rank** | 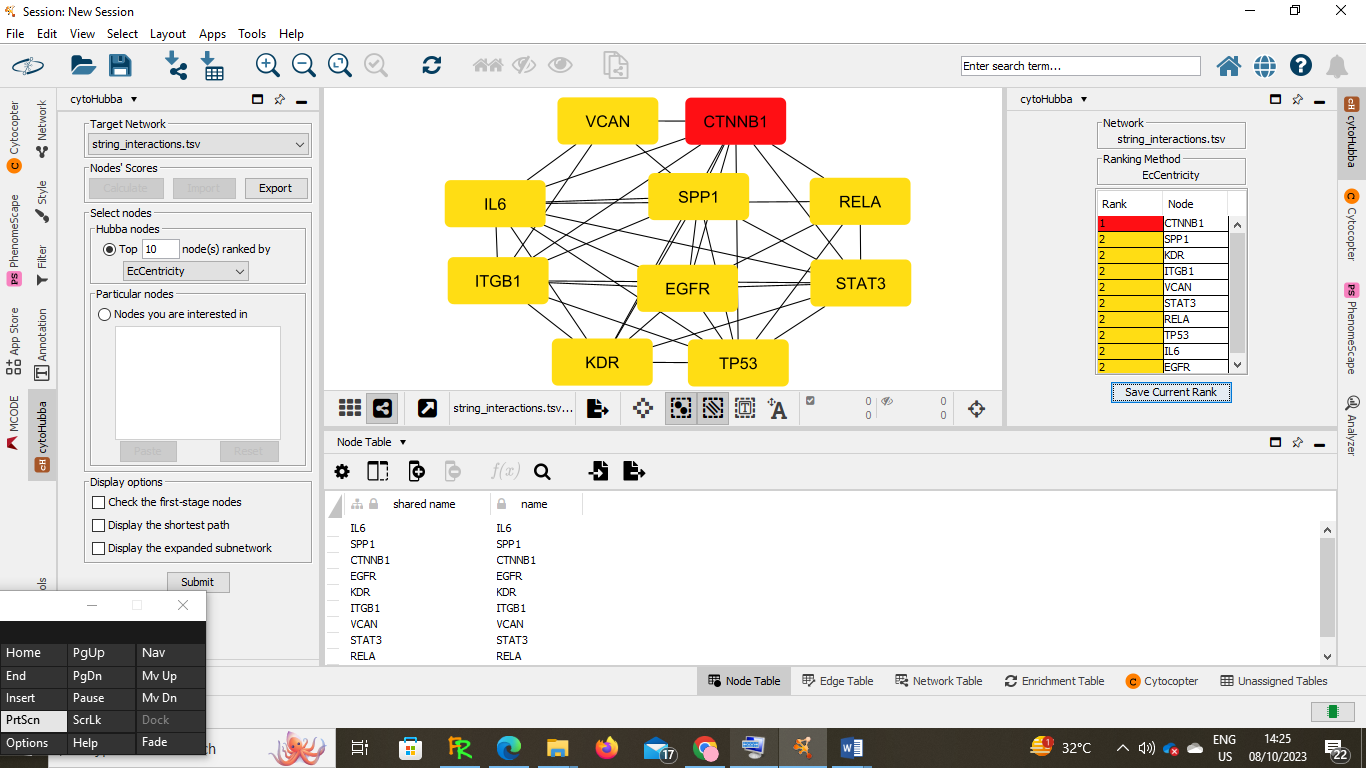 | 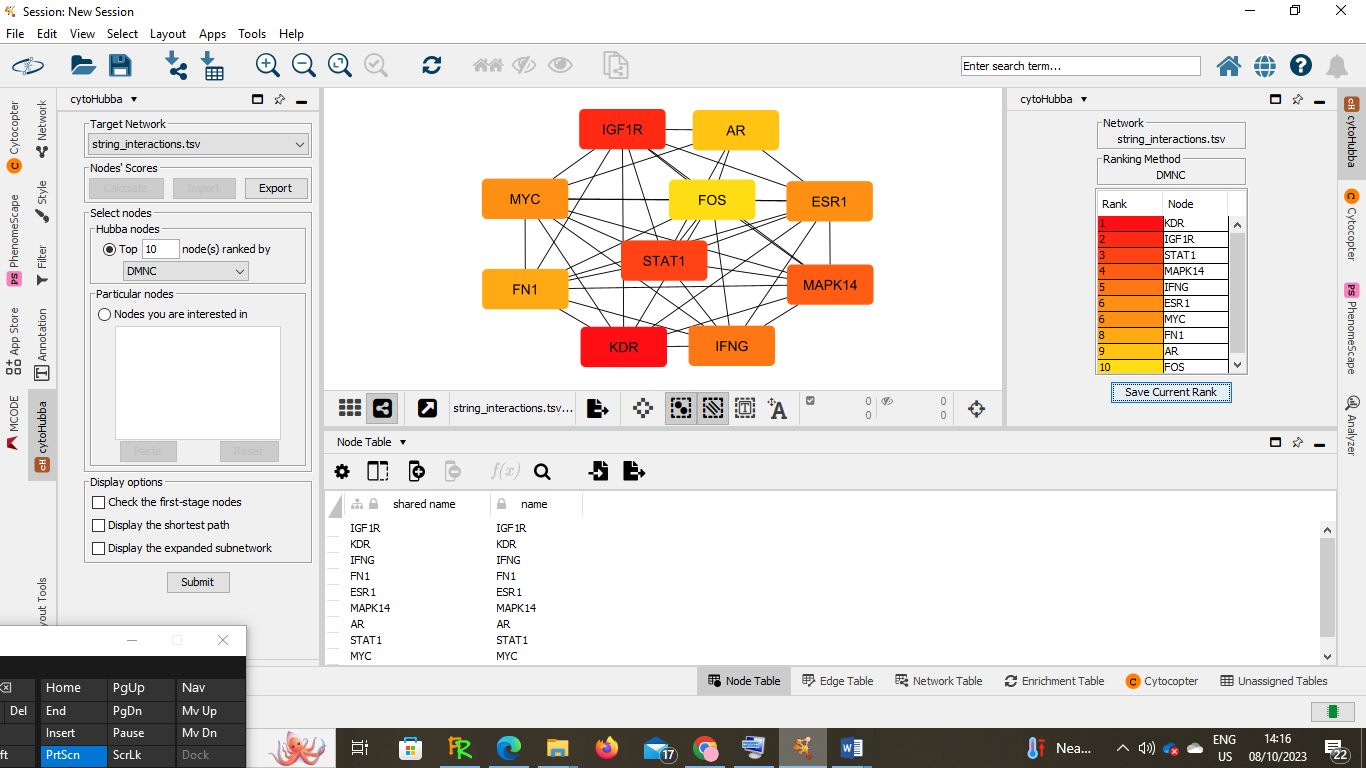 | 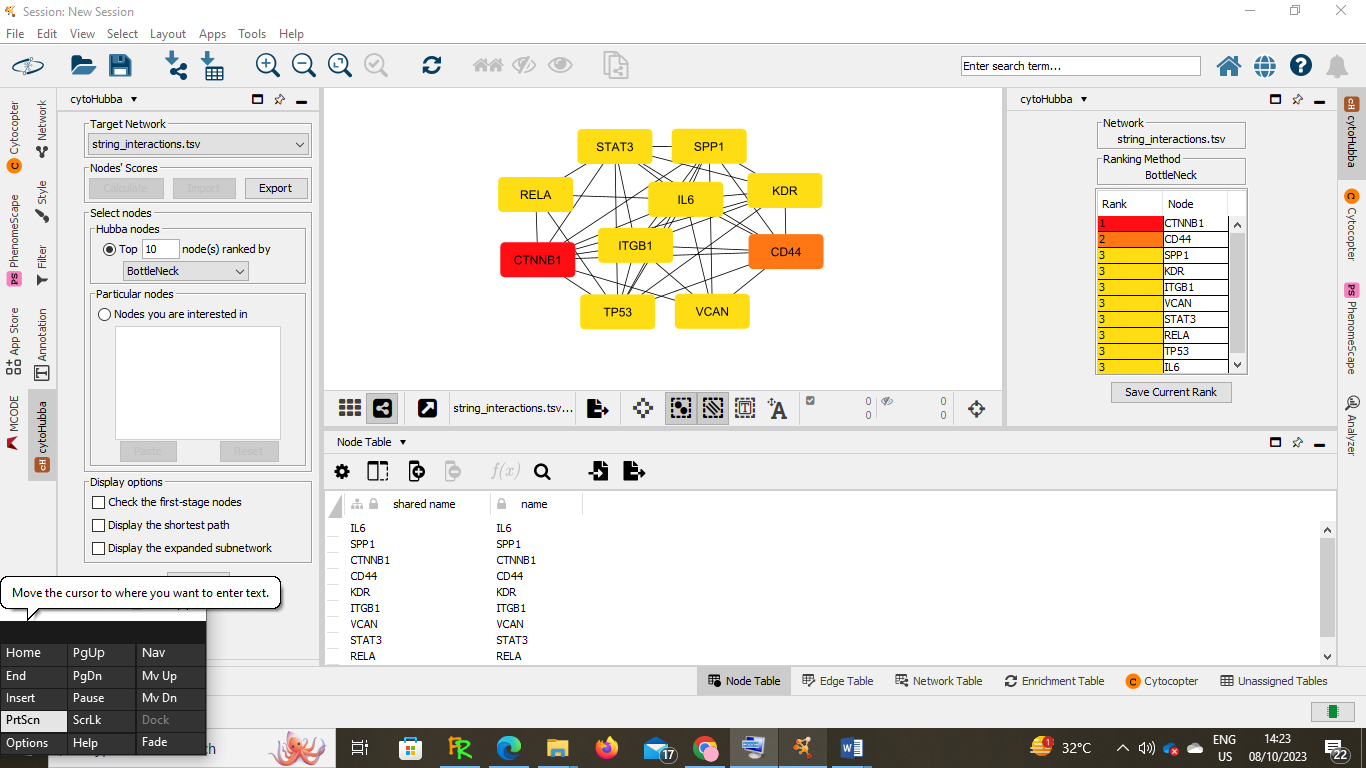 |
| **Network** | 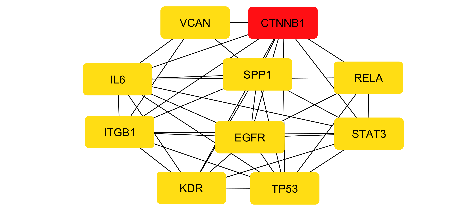 | 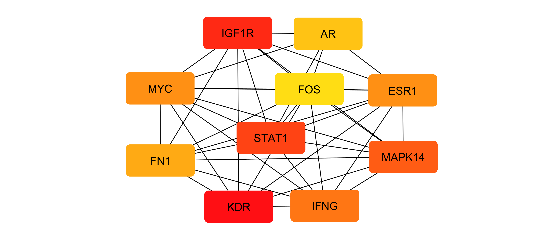 | 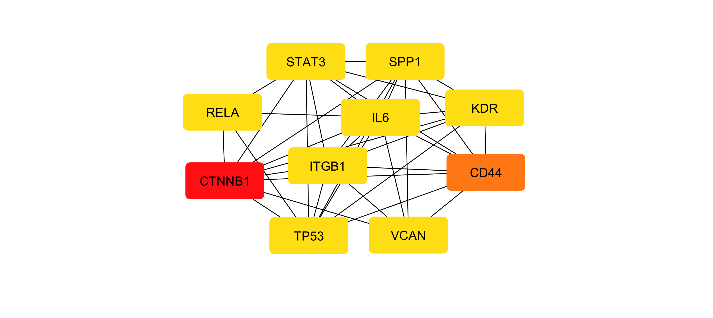 |
| **Method** | ***EPC*** | ***CC*** | ***Stress*** |
| **Rank** | 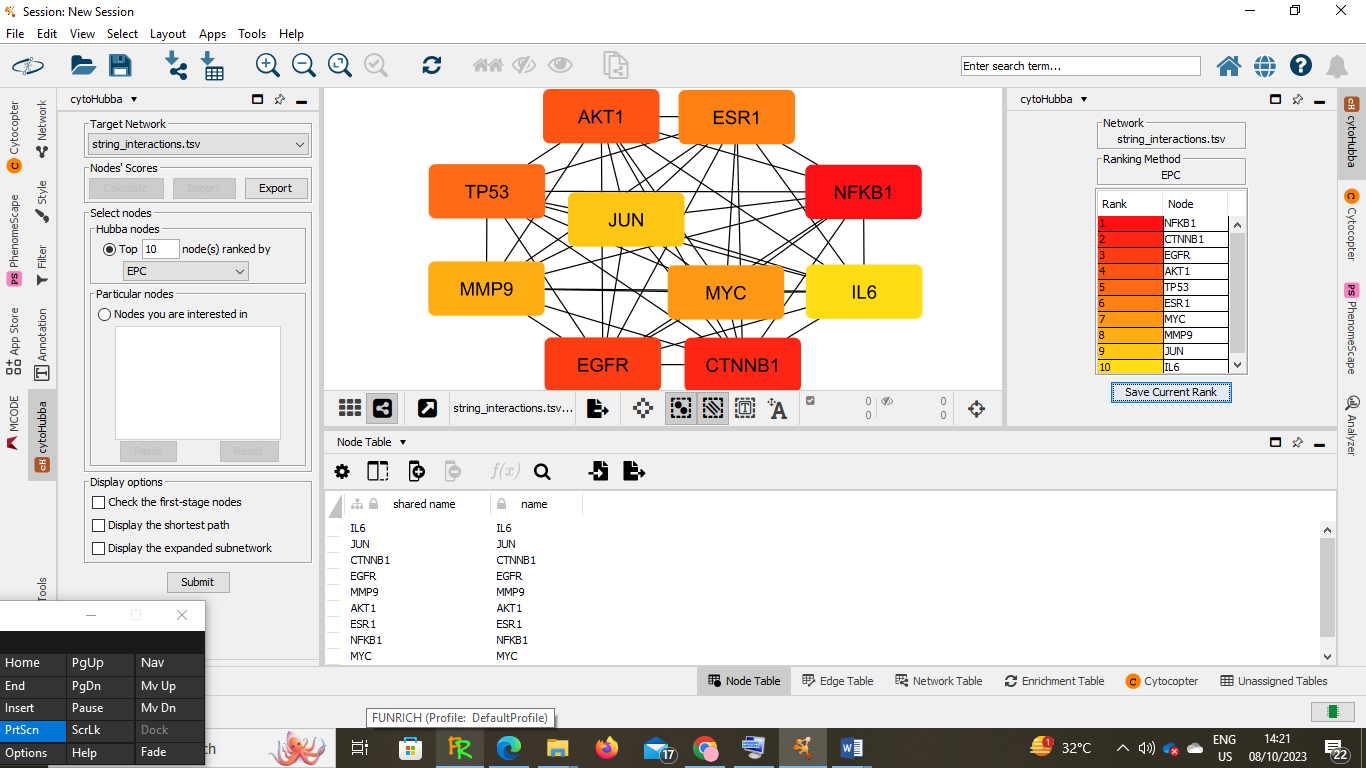 | 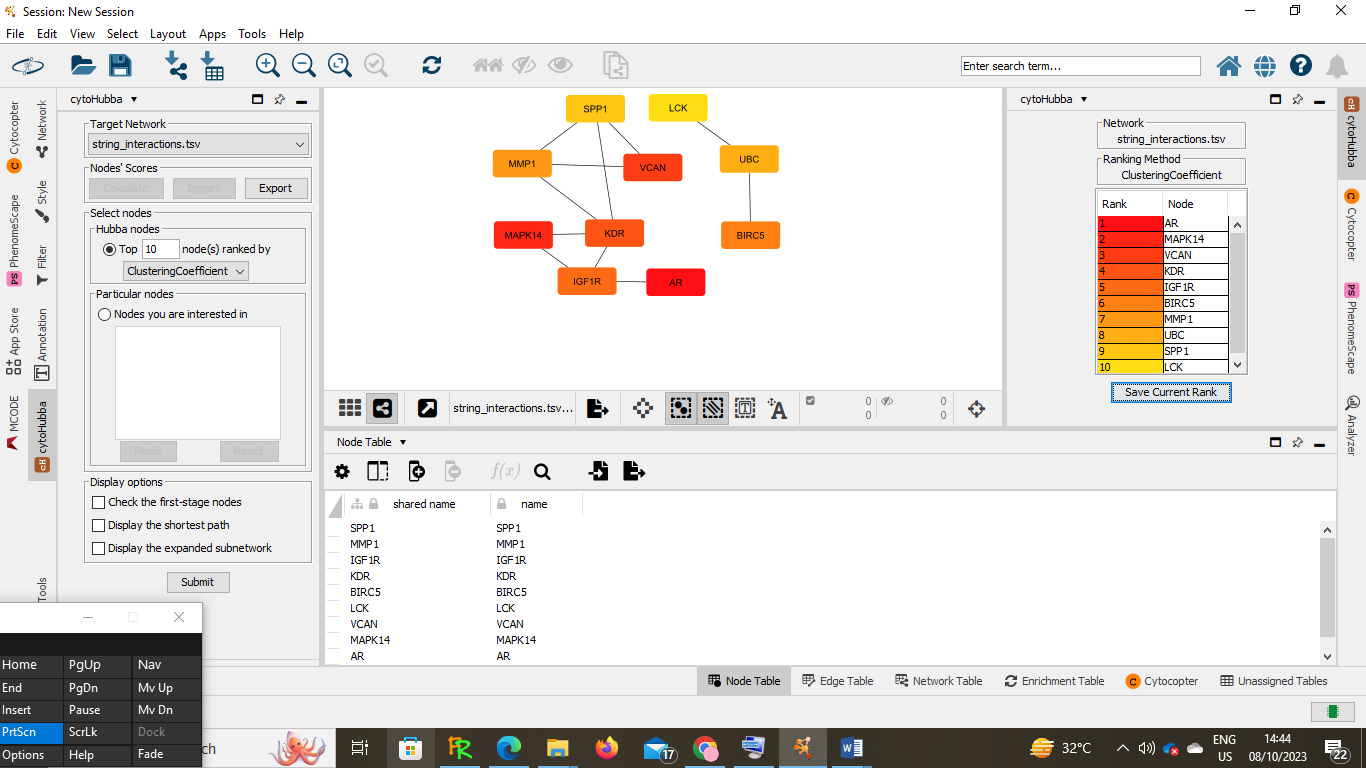 | 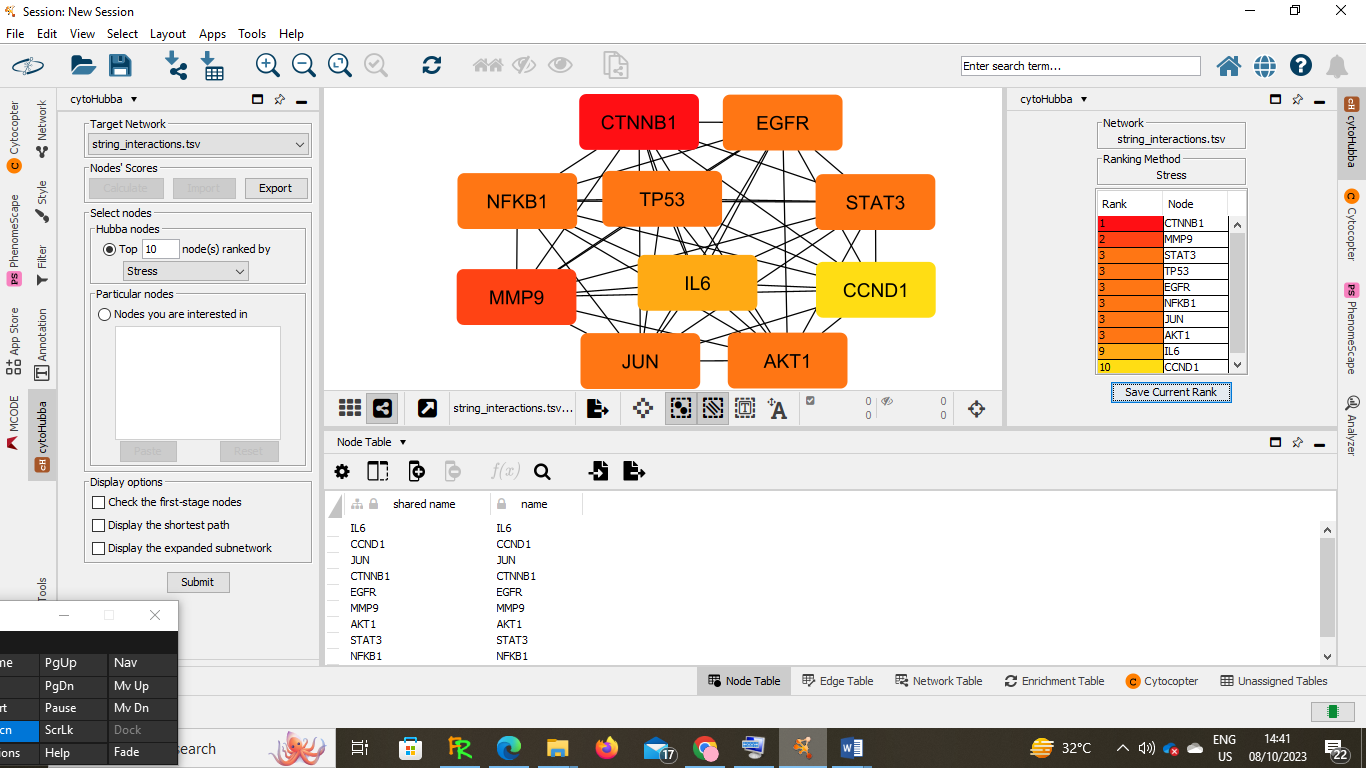 |
| **Network** | 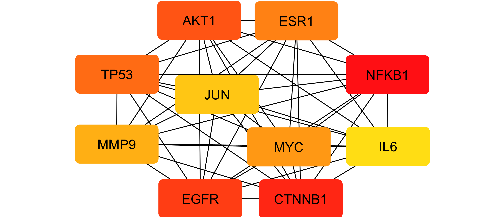 | 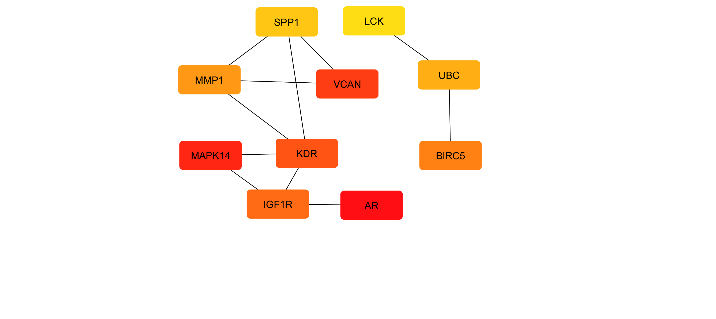 | 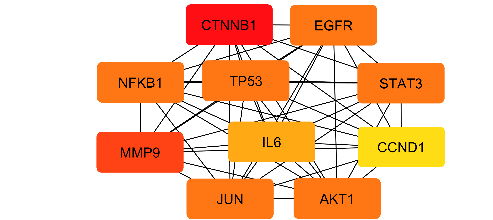 |

**Supplementary Table 3. Expression patterns for meta hub genes across diseases**.↑: Up-regulated; ↓: Down-regulated, ≈: Expressed, ×: Not expressed, -: Data not available, and ∆: Mutated.

| **#** | **Gene** | **Expression in T2DM** | **Expression in TNBC** | **Expression in OA** |
| --- | --- | --- | --- | --- |
| 1 | *IFNG* | ↑[148] | ≈[201] | ↑[202] |
| 2 | *CD44* | ↑[91] | ↑[142] | ↑[203] |
| 3 | *CTNNB1* | ≈[73] | ↑[75] | ↑[204] |
| 4 | *TP53* | ↑[205] | ∆[206] | ↑[207] |
| 5 | *CCND1* | ↑[208] | ↓[209] | ↑[210] |
| 6 | *EGFR* | ≈[211] | ↑[75] | ↓[212] |
| 7 | *ESR1* | ≈[213] | × | ≈[43] |
| 8 | *STAT3* | ≈[214] | ↑[96] | ↑[215] |
| 9 | *JUN* | ↑ | ≈[216] ↓[217] | ↑[218] |
| 10 | *MYC* | ↑ | ↑[219] | ↓[220] |
| 11 | *MMP1* | ↑[84] | ↑[83] | ↑[221] |
| 12 | *MMP9* | ↑[222] | ↑[223] | ↑[224] |
| 13 | *FOS* | ↑ | ↓[217] | ↑[225] |
| 14 | *KRAS* | - | ≈∆[226] | - |
| 15 | *AKT1* | ≈[227] | ↑ | ≈[67] |
| 16 | *NFKB1* | ↑[228] | ↑[229] | ↑[230] |
| 17 | *MMP2* | ↑[222] | ≈[231] | ≈[224] |

**Supplementary Table 4. Meta hub gene redundancy list.** The number of times each meta hub gene was identified as a hub gene in published literature is identified.

| **Sr. No.** | **Gene** | **Redundancy in T2DM**  **Hub Genes List** | **Redundancy in OA**  **Hub Genes List** | **Redundancy in TNBC Hub Genes List** |
| --- | --- | --- | --- | --- |
| 1 | *IFNG* | 1 | 1 | 1 |
| 2 | *CD44* | 1 | 3 | 1 |
| 3 | *CTNNB1* | 1 | 1 | 3 |
| 4 | *TP53* | 1 | 2 | 2 |
| 5 | *CCND1* | 3 | 1 | 3 |
| 6 | *EGFR* | 10 | 4 | 10 |
| 7 | *ESR1* | 4 | 2 | 12 |
| 8 | *STAT3* | 1 | 1 | 3 |
| 9 | *JUN* | 2 | 19 | 3 |
| 10 | *MYC* | 1 | 10 | 3 |
| 11 | *MMP1* | 1 | 1 | 2 |
| 12 | *MMP9* | 3 | 6 | 2 |
| 13 | *FOS* | 4 | 3 | 1 |
| 14 | *KRAS* | 1 | 1 | 1 |
| 15 | *AKT1* | 2 | 3 | 1 |
| 16 | *NFKB1* | 1 | 1 | 2 |
| 17 | *MMP2* | 1 | 5 | 1 |

**Supplementary Table 5. Meta hub gene- disease association.** Abbreviations: T2DM- type 2 diabetes mellitus; OA- osteoarthritis; BC- breast cancer.

| **Sr. No.** | **Meta Hub Genes** | **T2DM** | | **OA** | | **BC** | |
| --- | --- | --- | --- | --- | --- | --- | --- |
|  |  | ***Z Score*** | ***Confidence*** | ***Z Score*** | ***Confidence*** | ***Z Score*** | ***Confidence*** |
| 1 | *AKT1* | 7.4 | ★★★★ | 5.5 | ★★★ | 7.9 | ★★★★ |
| 2 | *NFKB1* | 5.9 | ★★★ | 5.1 | ★★★ | 6.3 | ★★★★ |
| 3 | *IFNG* | 6.2 | ★★★★ | 5.5 | ★★★ | 6.6 | ★★★★ |
| 4 | *CTNNB1* | 5.4 | ★★★ | 5.4 | ★★★ | 7.3 | ★★★★ |
| 5 | *EGFR* | 4.8 | ★★★ | 3.7 | ★★ | 8.0 | ★★★★ |
| 6 | *MMP9* | 5.5 | ★★★ | 5.8 | ★★★ | 6.9 | ★★★★ |
| 7 | *CD44* | 4.5 | ★★★ | 5.3 | ★★★ | 7.2 | ★★★★ |
| 8 | *MMP1* | 4.1 | ★★★ | 6.4 | ★★★★ | 5.5 | ★★★ |
| 9 | *STAT3* | 5.9 | ★★★ | 4.9 | ★★★ | 7.1 | ★★★★ |
